# Analyses of the Global Multilocus Genotypes of the Human Pathogenic Yeast *Candida tropicalis*

**DOI:** 10.3389/fmicb.2019.00900

**Published:** 2019-04-26

**Authors:** Jin-Yan Wu, Duan-Yong Zhou, Ying Zhang, Fei Mi, Jianping Xu

**Affiliations:** ^1^Public Research Laboratory, Hainan Medical University, Haikou, China; ^2^Laboratory for Conservation and Utilization of Bio-Resources and Key Laboratory for Microbial Resources of the Ministry of Education, Yunnan University, Kunming, China; ^3^Research Institute of Nutrition and Food Science, Kunming Medical University, Kunming, China; ^4^Department of Biology, McMaster University, Hamilton, ON, Canada

**Keywords:** multilocus sequence typing, genotype sharing, geographic pattern, genetic clusters, yeast, geographic distribution

## Abstract

*Candida tropicalis* is a globally distributed human pathogenic yeast, especially prevalent in tropical and sub-tropical regions. Over the last several decades, a large number of studies have been published on the genetic diversity and molecular epidemiology of *C. tropicalis* from different parts of the world. However, the global pattern of genetic variation remains largely unknown. Here we analyzed the published multilocus sequence data at six loci for 876 isolates from 16 countries representing five continents. Our results showed that 280 of the 2677 (10.5%) analyzed nucleotides were polymorphic, resulting in a mean of 82 (a range of 38–150) genotypes per locus and a total of 633 combined diploid sequence types (DSTs). Among these, 93 combined DSTs were shared by 336 strains, including 10 by strains from different continents. Analysis of Molecular Variance (AMOVA) showed that 89% of the observed genetic variations were found within regional and national populations while < 10% was due to among-country separations. Pairwise geographic population analyses showed overall low but statistically significant genetic differentiation between most geographic populations, with the Singaporean and Indian populations being the most distinct from other populations. However, the Mantel test showed no significant correlation between genetic distance and geographic distance among the geographic populations. Consistent with high genetic variation within and limited variations among geographic populations, results from STRUCTURE analyses showed that the 876 isolates could be grouped into 15 genetic clusters, with each cluster having a broad geographic distribution. Together, our results suggest frequent gene flows among certain regional, national, and continental populations of *C. tropicalis*, resulting in abundant regional and national genetic diversities of this important human fungal pathogen.

## Introduction

*Candida tropicalis* is an ascomycete yeast and an important opportunistic human pathogen (Falagas et al., 2010; Zuza-Alves et al., 2017). It’s broadly distributed in a diversity of ecological niches such as soil, aquatic environments, plant materials, and animals, including humans (Vogel et al., 2007; Lord et al., 2010; Lachance et al., 2011; Brilhante et al., 2013, 2015; Cordeiro et al., 2013). In human hosts, *C. tropicalis* is commonly found in the oral cavity and on certain parts of the skin and mucosal surfaces as a commensal organism. With an increasing number of immunocompromised patients due to cancer treatment, HIV infection, and/or organ transplantation, *C. tropicalis* has emerged as a major opportunistic pathogen that can cause serious invasive infections such as endocarditis and the bloodstream and urinary tract infections, resulting in significant morbidity and mortality (Falagas et al., 2010; Fesharaki et al., 2013; Dong et al., 2015; Zuza-Alves et al., 2017; Diba et al., 2018). In certain geographic regions, *C. tropicalis* is ranked as the first or second most prevalent invasive pathogenic yeast species (Adhikary and Joshi, 2011; Dóczi et al., 2012; Wang et al., 2013; Morii et al., 2014; Pahwa et al., 2014; Arrua et al., 2015; Al-Obaid et al., 2017). However, despite its global medical significance, relatively little is known about its global patterns of genetic variation.

Over the past three decades, several molecular methods have been used to identify genotypes and examine the relationships among strains and populations of human pathogenic yeasts (Xu, 2005; Lachance et al., 2011; Zuza-Alves et al., 2017). These included multilocus isozyme electrophoresis (e.g., Brandt et al., 1993), electrophoretic karyotyping (e.g., Fries et al., 1996), PCR fingerprinting (e.g., Xu et al., 2000a), amplified fragment length polymorphisms (e.g., Duarte-Escalante et al., 2013), simple sequence repeats (e.g., Field et al., 1996), and multilocus sequence typing (MLST) (e.g., Xu et al., 2000b; Tavanti et al., 2005; Afsarian et al., 2015). For *C. tropicalis*, the emerging consensus since 2005 for strain typing is MLST, based on the single nucleotide polymorphisms (SNPs) at six gene fragments (Tavanti et al., 2005). These marker loci were *ICL1, MDR1, SAPT2, SAPT4, XYR1*, and *ZWF1a* and they were chosen for MLST of *C. tropicalis* for several reasons, including: (i) the high success rates of PCR amplification and sequencing using the same primers; (ii) their high-levels of single nucleotide polymorphisms (SNPs) within and among strains; and (iii) when sequences at the six loci are combined, their high genotypic discriminating power among strains (Tavanti et al., 2005). Indeed, these MLST markers have not only been used to analyze the relationships among strains from different hosts and wards within hospitals, but also to monitor strain maintenance, replacement, and microevolution within individual human hosts (Chen et al., 2009; Wu et al., 2012; Magri et al., 2013). Furthermore, the establishment of the MLST database for *C. tropicalis* has facilitated the comparisons of strains and populations from different laboratories and studies (e.g., Wu et al., 2017; Scordino et al., 2018). As of the end of May 2018, the MLST database for *C. tropicalis* includes DNA sequence information at these six loci for 876 isolates. However, the global genetic structure of *C. tropicalis* populations remains poorly known.

The objective of this study was to investigate the global patterns of genetic variation by analyzing the published MLST sequence data of *C. tropicalis* from around the world. We were specifically interested in the geographic patterns of DNA sequence variation and in where some of our knowledge gaps might be. Because *C. tropicalis* is commonly found in the environment (Odds, 1988; Vogel et al., 2007; Lachance et al., 2011; Carvalho et al., 2014) and that most hosts likely acquire their commensal strains from their immediate environment, we hypothesized that populations of *C. tropicalis* from different countries and continents should be genetically different from each other and that geographic distance should contribute a significantly to the overall genetic differentiation among geographic populations.

## Materials and Methods

All DNA sequence data as well as the meta-data associated with each strain were retrieved from the *Candida tropicalis* multilocus sequence typing database^1^. The species identification, DNA extraction, PCR amplification, and DNA sequencing for all strains were described in the original publications reporting the strains in the database. The database was curated by Dr. Frank Odds and his colleagues and the retrieval and use of the data were approved and supported by Dr. Frank Odds. For each strain, the deposited DNA sequence at each of the six loci was separately downloaded. All the retrieved sequences for each locus were aligned and imported into GenAlEx 6.5 (Peakall and Smouse, 2012) for analyses. Since *C. tropicalis* is a diploid and heterozygous nucleotide sites have been frequently found, our analyses of the patterns of DNA sequence variation follow those for diploids. The number of polymorphic nucleotide sites and the number of genotypes at each locus were then obtained through this program, following those described in an earlier study (Wu et al., 2017).

To determine strain relationships, we combined the DNA sequences at the six loci and analyzed the concatenated sequences through cluster analysis using UPGMA (unweighted pair group method using their arithmetic averages) of the MEGA Version 6 software (Tamura et al., 2013). To analyze multilocus genotype distributions and patterns of genetic variations, the concatenated DNA sequences from all six loci for all strains were imported into GenAlEx6.5. The analysis of molecular variance (AMOVA) was conducted at three geographic levels depending on the samples. Specifically, due to the availability and relatively large sample sizes from multiple regions within China, our first-level analysis estimated the within- and among-regional populations to the overall genetic variation in the Chinese *C. tropicalis* sample. The second and third-level analyses were conducted together and estimated the relative contributions of within countries, between countries within continents, and among continents to the overall genetic variation. All AMOVA analyses were conducted using GenAlEx6.5. To further investigate the relationships between specific geographic populations, we calculated pairwise PhiPT values between pairs of regional populations within China and pairs of national populations. In these analyses, only samples with a sample size > 10 were included. Statistical significance of the observed contributions at various geographic levels to the overall patterns of genetic variation was obtained by 1000 randomizations. In addition, the potential relationship between geographic distance and genetic differentiation was estimated using the Mantel test. The detailed instructions for AMOVA, pairwise PhiPT, and Mantel tests can be found in the GenAlEx program manual (Peakall and Smouse, 2012).

Aside from the above population genetic analyses, we also estimated the putative number of genetic clusters K in the global sample of *C. tropicalis* using the program STRUCTURE version 2.3.4 (Pritchard et al., 2000). STRUCTURE implements a clustering algorithm based on a Bayesian Monte Carlo Markov Chain (MCMC) approach to assign individuals into K distinct populations. Using the admixture model, 10 replicated runs of *K* = 1–20 were carried out after a burn-in period of 100,000 generations followed by a run length of 1,000,000 generations. The number of genetically homogeneous clusters (K) was identified by following the method developed by Evanno et al. (2005). STRUCTURE 2.3.4 was also used to identify the assignment of individual isolates to specific clusters (K).

## Results

Up to the end of May 2018, a total 876 isolates were deposited in the *C. tropicalis* MLST database. The metadata and DNA sequences at the six MLST loci of all 876 strains were retrieved from the database. Below we summarize the observed data and the results of our analyses.

### Geographic and Ecological Distributions of Strains in the MLST Database

The geographic distributions of these strains in the *C. tropicalis* MLST database are shown in Table 1. These strains came from broad geographic areas, representing 16 countries on five continents. At the continental level, the vast majority of the 876 strains were from Asia (∼74%), followed by Europe (∼16%), South America (∼7%), North America (∼2%), and Australia (0.8%). There was no strain from Africa in the database and over 90% of the countries in the world were not represented. At the country level, 67.58% of the global strains in the MLST database were from China; followed by the United Kingdom (∼14%); Brazil (∼5%); India (2.9%); South Korea (2.5%); the United States (1.9%); Singapore (1.5%); Belgium (1.1%); Colombia (1%); Australia (0.8%); Argentina (0.46%); Germany and Netherlands (0.34% each); and Sweden, Greece, and Spain (one strain from each of the three countries; 0.11%). Within an individual country, China not only contributed the largest number of strains but also that the strains were from broad geographic regions within the country, from tropical Hainan province to temperate Heilongjiang province.

**Table 1:** Distribution of the geographic populations of *Candida tropicalis* analyzed in this study.

Continent	Country	Province/State (City)	Sample size
Asia			652
	China		592
		Beijing	82
		Hainan	118
		Heilongjiang	14
		Jiangxi (Nanchang)	17
		Shanghai	52
		Guangdong (Shenzhen)	38
		Sichuan	11
		Tianjin	7
		Taiwan	253
	India		25
	South Korea	Gwanjiu	22
	Singapore		13
Europe			143
	Belgium	Antwerp	10
	Germany	Frankfurt	3
	Netherlands		3
	United Kingdom		124
		Scotland	12
		England (Leeds and London)	76
		Unspecified	36
	Sweden, Greece, Spain		3 (one per country)
North America	United States		17
Oceania	Australia		7
South America			59
	Argentina	Buenos Aires	4
	Colombia		9
	Brazil		46
		Campinas	27
		Recife	8
		São Paulo	11
Total			876


Among the 876 strains, 760 had information about their ecological niche or anatomic body site while the remaining 106 had no ecological niche or anatomic body site information (Supplementary Table 1). Among the 760 strains, three were from animals (from Belgium and Spain) and 757 were from humans. The human isolates came from several types of human tissues and organs, including the blood (287 strains total; from Argentina, Australia, Brazil, China, Colombia, India, Korea, Singapore, Netherlands, United Kingdom, and United States); feces (39 strains total; from China and United Kingdom); oropharynx (275 strains total; from Belgium, Brazil, China, Germany, India, Korea, United Kingdom, and United States); other sterile body sites (14 strains total, from Brazil, China, Colombia, Singapore, United Kingdom, and United States); skin and other superficial surfaces (40 strains total, from Belgium, China, Korea, and United Kingdom); urine (97 strains total, from China, Colombia, and United Kingdom); and vagina (58 strains total, from Belgium, Brazil, China, Sweden, United Kingdom, and United States).

### DNA Sequence Variation

We retrieved the DNA sequences at the six MLST loci (*ICL1, MDR1, SAPT2, SAPT4, XYR1*, and *ZWFa1*) for all 876 isolates. Information about these loci and the levels of DNA sequence polymorphisms within this global sample are summarized in Table 2. These six sequenced DNA fragments ranged in sizes from 370 to 525 bp and they are located on different scaffolds (Tavanti et al., 2005; Butler et al., 2009). Together, the six gene fragments cover a total of 2677 bp, representing < 0.02% of the entire genome. The haploid genome size of *C. tropicalis* is estimated at about 14.63 Mb, containing 6,441 genes (Butler et al., 2009). All six sequenced gene fragments were highly polymorphic in this global sample. Specifically, the percentages of nucleotides that are polymorphic in the global sample range from 7.11 to 16.41%, with an overall 10.46% (280/2677). At these six loci, there was no clear correlation between the length of sequenced fragment and the number of polymorphic nucleotide sites (Pearson correlation coefficient *R* = 0.0093; *p* = 0.986). Indeed, the fragment (i.e., *SAPT4*) with the highest number of SNPs (at 64) was the second shortest sequenced fragment, at 390 bp. At present, the reason for the different levels of polymorphisms among the sequenced gene fragments is not known but may be related to the different environmental and/or functional constraints that these genes have experienced during the evolution of *C. tropicalis*.

**Table 2:** Single nucleotide polymorphisms at the six loci in the global sample of *C. tropicalis.*

Gene name^a^	Chromosomal scaffold ID (Length of scaffold)^b^	Sequenced fragment length (location within scaffold)	Number of polymorphic nucleotide sites (%)	Number of diploid genotypes per locus
*ICL1*	NW_003020058.1	447 bp	33 (7.38%)	38
	(1,255,791 bp)	(959,343–959,789)		
*MDR1*	NW_003020055.1	425 bp	49 (11.53%)	150
	(2,216,334 bp)	(754,510–754,086)		
*SAPT2*	NW_003020038.1	525 bp	60 (11.43)	45
	(2,474,448 bp)	(1,573,960–1,574,484)		
*SAPT4*	NW_003020049.1	390 bp	64 (16.41%)	81
	(2,308,670 bp)	(2,274,975–2,274,586)		
*XYR1*	NW_003020040.1	370 bp	37 (10.00%)	131
	(419,327 bp)	(35,519–35,150)		
*ZWF*	NW_003020056.1	520 bp	37 (7.11%)	45
	(1,654,078 bp)	(515,841–516,360)		
Total		2677 bp	280 (10.46%)	633 (out of 876 strains)


*^a^*ICL1*, isocitrate lyase; *MDR1*, multidrug resistance protein; *SAPT2*, secreted aspartic protease 2; *SAPT4*, secreted aspartic protease 4; *XYR1*, D-xylose reductase I or II; *ZWF1a* Putative glucose-6- phosphate dehydrogenase (Tavanti et al., 2005). ^*b*^The GenBank accession number for this genome sequence is AAFN01000000. The relevant information about this genome can be accessed at: www.ncbi.nlm.nih.gov/bioproject/13675. The *C. tropicalis* genome is estimated to contain 6,441 genes and has a genome size of 14.63 Mb. The above six scaffolds are among 23 chromosomal scaffolds assembled for this *C. tropicalis* genome.*

The 280 SNPs resulted in the identification of a large number of genotypes at both the individual locus level as well as the combined six loci (Table 2). At the individual locus level, the number of genotypes ranged from 38 (locus *ICL1*) to 150 (locus *MDR1*), with a mean of 81.67. Similar to that in SNP density, the number of genotypes per locus was not significantly correlated with either fragment length or SNP density. For example, the Pearson correlation coefficient between the number of SNPs and number of genotypes at the six loci was 0.042, with a *P*-value of 0.937. In contrast to our expectation, there was a negative correlation between the sequenced fragment length and the number of genotypes among the six loci (Pearson correlation coefficient = -0.688). However, this seemingly negative correlation was statistically insignificant (*P* = 0.132). Regardless, these results are consistent with the distinctiveness of these six loci in the *C. tropicalis* genome (Table 2) and that it’s often difficult to predict the level of genotypic polymorphism based on either the fragment length and/or SNP density.

### Distribution of the Combined Diploid Sequence Types

The relationships among all 876 strains are shown in Supplementary Figure 1. Together, the combined analyses of all six gene fragments identified a total of 633 multilocus genotypes out of the 876 strains (Supplementary Table 1). Among these 633 combined genotypes, 93 were shared by a total of 336 strains (Table 3). The remaining 540 multilocus genotypes were each represented by only one strain. Of the 93 shared multilocus genotypes, the majority (56; 60%) were only found within one region of one country each. The remaining 37 multilocus genotypes showed different distributions: 23 were shared by strains from different regions within a country, four were shared among countries within a continent, and 10 were shared among continents. Of these 37 multilocus genotypes, 17 were also shared among strains within a region of a country (Table 3).

**Table 3:** Shared multilocus genotypes of *Candida tropicalis* in the MLST database.

Multilocus genotype number	Number of strains	Source of strains (Number of strains)	Shared within a region	Shared between regions within a country	Shared between countries within a continent	Shared between continents
3	2	Belgium (2)	+	–	–	–
6	2	CA, United States (2)	+	–	–	–
7	2	CA, United States (2)	+	-	-	-
12	2	Belgium (1) United States (1)	–	–	–	+
13	7	Belgium (1) Greece (1) London, United Kingdom (5)	+	–	+	–
15	2	Frankfurt, Germany (2)	+	–	–	–
18	4	London, United Kingdom (4)	+	–	–	–
23	3	Hainan, China (1) Netherlands (1) Unknown (1)	–	–	–	+
27	2	Taiwan, China (1) United States (1)	–	–	–	+
31	4	Leeds, United Kingdom (1) Aberdeen, United Kingdom (1) United Kingdom (2)	-/+	+	–	–
32	2	Aberdeen, United Kingdom (1) United Kingdom (1)	-/+	-/+	–	–
45	3	Taiwan, China (1) United Kingdom (2)	-/+	-/+	–	+
69	2	United Kingdom (2)	-/+	-/+	–	–
80	3	Recife, Brazil (2) United States (1)	+	–	–	+
83	3	London, United Kingdom (3)	+	–	–	–
90	8	Recife, Brazil (3) Colombia (2) Taiwan, China (3)	+	–	+	+
92	9	London, United Kingdom (9)	+	–	–	–
93	3	London, United Kingdom (3)	+	–	–	–
94	2	London, United Kingdom (2)	+	–	–	–
96	2	London, United Kingdom (2)	+	–	–	–
98	9	Colombia (2) Taiwan, China (7)	+	–	–	+
99	2	Beijing, China (1) Colombia (1)	–	–	–	+
100	2	Colombia (2)	+	–	–	–
103	2	London, United Kingdom (2)	+	–	–	–
106	3	London, United Kingdom (3)	+	–	–	–
114	5	Harbin, China (1) Shenzhen, China (1) London, United Kingdom (3)	+	+	–	+
120	2	London, United Kingdom (2)	+	–	–	–
134	7	Taiwan, China (7)	+	–	–	–
139	4	Shenzhen, China (1) Taiwan, China (3)	+	+	–	–
140	25	Taiwan, China (25)	+	–	–	–
144	2	Taiwan, China (2)	+	–	–	–
149	17	Hainan, China (5) Taiwan, China (12)	+	+	–	–
164	11	Tianjin, China (1) Taiwan, China (10)	+	+	–	–
165	2	Taiwan, China (2)	+	–	–	
168	4	Taiwan, China (4)	+	–	–	–
169	8	Harbin, China (1) Beijing, China (1) Shenzhen, China (3) Taiwan, China (3)	+	+	–	–
170	2	Taiwan, China (2)	+	–	–	–
172	2	Taiwan, China (1) Scotland, United Kingdom (1)	–	–	–	+
179	2	Taiwan, China (2)	+	–	–	–
183	2	Taiwan, China (2)	+	–	–	–
187	2	Taiwan, China (2)	+	–	–	–
188	2	Taiwan, China (2)	+	–	–	–
191	2	Taiwan, China (2)	+	–	–	–
197	2	Hainan, China (1) Taiwan, China (1)	–	+	–	–
200	2	Taiwan, China (2)	+	–	–	–
206	2	India (2)	+	–	–	–
214	4	India (4)	+	–	–	–
218	2	India (2)	+	–	–	–
220	2	India (2)	+	–	–	–
226	2	Taiwan, China (2)	+	–	–	–
227	3	Taiwan, China (3)	+	–	–	–
238	2	Campinas, Brazil (2)	+	–	–	–
246	2	Campinas, Brazil (2)	+	–	–	–
277	2	Beijing, China (2)	+	–	–	–
278	2	Beijing, China (2)	+	–	–	–
279	5	Beijing, China (5)	+	–	–	–
321	3	Harbin, China (1) Chengdu, China (1) Shenzhen, China (1)	–	+	–	–
322	3	Harbin, China (3)	+	–	–	–
328	2	Harbin, China (2)	+	–	–	–
330	5	Chengdu, China (2) Hainan, China (1) Shenzhen, China (2)	+	+	–	–
331	7	Chengdu, China (1) Beijing, China (2) Hainan, China (4)	+	+	–	–
332	2	Chengdu, China (1) Shenzhen, China (1)	–	+	–	–
333	4	Chengdu, China (1) Hainan, China (1) Shenzhen, China (2)	+	+	–	–
336	2	Chengdu, China (1) Hainan, China (1)	–	+	–	–
343	2	Tianjin, China (1) Hainan, China (1)	–	+	–	–
346	5	Beijing, China (1) Hainan, China (3) Shenzhen, China (1)	+	+	–	–
348	2	Beijing, China (1) Hainan, China (1)	–	+	–	–
351	2	Beijing, China (1) Hainan, China (1)	–	+	–	–
356	2	Beijing, China (2)	+	–	–	–
374	2	Hainan, China (1) Taiwan, China (1)	–	+	–	–
394	9	Hainan, China (7) Shenzhen, China (1) Gwanju, Korea (1)	+	+	+	–
420	2	Hainan (1) Shenzhen (1)	–	+	–	–
426	2	Gwanju, Korea (1) Shenzhen (1)	–	–	+	–
427	2	Hainan, China (2)	+	–	–	–
430	4	Hainan, China (4)	+	–	–	–
432	2	Hainan, China (2)	+	–	–	–
437	2	Hainan, China (1) Shenzhen, China (1)	–	+	–	–
465	2	Hainan, China (2)	+	–	–	–
489	3	Hainan, China (1) Shenzhen, China (2)	+	+	–	–
490	2	Hainan, China (2)	+	–	–	–
499	2	Singapore (2)	+	–	–	–
504	2	Shanghai, China (2)	+	–	–	–
507	13	Shanghai, China (13)	+	–	–	–
508	4	Shanghai, China (4)	+	–	–	–
520	4	Shanghai, China (4)	+	–	–	–
522	3	Shanghai, China (2) Shenzhen, China (1)	+	+	–	–
525	2	Shanghai, China (2)	+	–	–	–
532	3	Shanghai, China (1) Shenzhen, China (2)	+	+	–	–
536	2	Singapore (2)	+	–	–	–
606	3	Nanchang, China (3)	+	–	–	–
607	2	Nanchang, China (2)	+	–	–	–
609	3	Nanchang, China (3)	+	–	–	–
723	3	Shenzhen, China (3)	+	–	–	–


*+, evidence of sharing; –, no evidence of sharing in the present sample; -/+, unknown due to uncertain status of some strains at the regional level.*

Among the 93 shared multilocus genotypes, the most frequent (genotype #140) was shared by 25 strains, all of which were from Taiwan (Table 3). The second most frequent multilocus genotype (genotype #149) was shared by 17 strains with five from Hainan and 12 from Taiwan, two geographically isolated islands off the south and east coasts of Mainland China respectively. The third most frequently shared multilocus genotype (genotype# 507) had 13 strains, all of which were from Shanghai. The fourth and fifth most frequently shared were multilocus genotypes #164 and #169 that contained strains from different regions of Mainland China and Taiwan. These results are consistent with clonal dispersal of several genotypes of *C. tropicalis* within and across diverse regions in China.

The 56 multilocus genotypes exclusively shared among strains within a region were distributed in all continents, including most of the countries represented in the database: Belgium, Brazil, China, Colombia, Germany, India, Singapore, United Kingdom, and the United States (Table 3). Most multilocus genotypes that were shared among regions within a country involved strains from China. This result was likely due to the fact that China was the most intensively sampled country for *C. tropicalis* in terms of the diversity of geographic locations and the number of strains within a country. In contrast to the skewed distribution at the local and within a country-level, the multilocus genotypes shared among countries were geographically more broadly represented, with multilocus genotype #13 from Europe, #90 from South America, and #394 and #426 from Asia.

Ten multilocus genotypes were shared by strains from different continents. Genotype #12 was shared between Belgium and the United States; genotype #23 between China and the Netherlands; genotype #27 between China and the United States; genotypes #45, #114, #172 between China and the United Kingdom; genotype #80 between Brazil and the United States; genotype #90 among Brazil, Colombia, and China, and genotypes #98 and #99 between Colombia and China. Though countries from Asia, Europe, South America and North America were all represented here, there was a bias for strains from China, with eight of the 10 multilocus genotypes having representative strains from China. Again, the bias was most likely related to the differences in sample sizes in the *C. tropicalis* MLST database.

Together, the above results suggest that short-, medium-, and long- distance dispersals were all possible for *C. tropicalis* strains.

### Geographic Contributions to Genetic Variation

#### Analysis of Molecular Variance

The sharing of multilocus genotypes between regional, national and continental populations suggests that gene flow might be common in *C. tropicalis*. To quantify the effects of geographic separation at regional, national and continental levels on the observed genetic variation, we conducted an analysis of molecular variance (AMOVA). At the global level, our analyses revealed that the majority (89%) of the SNP variations was found within individual countries while that among countries contributed the remaining 11% (Table 4). Interestingly, there is almost no contribution at the continental level beyond that at the national level. A similar trend was observed for the samples from China where the majority of the observed genetic variation (94%) was found within the regional populations and the remaining 6% was due to between-regional (i.e., provincial) populations. Though the contributions from among regional populations within China (6%) and among national populations at the global level (11%) were relatively low, such contributions were statistically significant (*p*-values < 0.001 in both cases). Together, these results are consistent with an overall low but statistically significant differentiation among regional populations within China and among national populations in the world.

**Table 4:** Analyses of molecular variance (AMOVA) at different geographic levels.

Source	df	SS	MS	Est. Var.	%
Among continents	3	202.692	67.564	0.000	0%
Among countries	5	242.623	48.525	1.310	11%
Within countries	842	8650.769	10.274	10.274	89%
Total	850	9096.085		11.584	100%


*Only countries with more than 10 isolates were included in this analysis.*

#### Differences Between National Populations

We further investigated the extent of genetic differentiation between pairs of national populations. The summary results among national populations are shown in Table 5. Among these, the Singapore sample was genetically the most differentiated from others (all with PhiPT values greater than 20%), followed by those between India and the remaining countries (all with PhiPT values between 12 and 15%). Between other pairs of national samples, their PhiPT values were about 10% or less. Statistically speaking, the only pairs of samples that were not significantly differentiated were those between Belgium and the United Kingdom, between Belgium and the United States, and between Brazil and the United States. The remaining pairs of samples were all statistically significantly differentiated at *p* < 0.05 (Table 5).

**Table 5:** Genetic differentiation between pairs of geographic populations of *C. tropicalis* from different countries.

	China	India	Korea	Singapore	Belgium	United Kingdom	United States	Brazil
China		0.001	0.001	0.001	0.013	0.001	0.001	0.001
India	0.147		0.001	0.001	0.011	0.001	0.003	0.001
Korea	0.071	0.123		0.001	0.007	0.001	0.002	0.002
Singapore	**0.203**	**0.269**	**0.309**		0.001	0.001	0.001	0.001
Belgium	0.053	0.126	0.081	**0.392**		0.336	0.028	0.069
UK	0.044	0.146	0.087	**0.239**	0.003		0.002	0.001
USA	0.066	0.144	0.106	**0.385**	0.102	0.062		0.065
Brazil	0.080	0.138	0.086	**0.249**	0.060	0.042	0.027	


*Only countries with more than 10 isolates were included in this analysis. PhiPT values are below diagonal. Probability, P(rand > = data) based on 999 permutations is shown above diagonal. Bolded numbers show the geographic population most distinct from other geographic populations.*

#### Differences Between Regional Populations Within Countries

The extent of genetic differentiation between pairs of regional populations were estimated for those in three countries (Brazil, United Kingdom, and China). Among the three regional samples in Brazil (Campinas, Recife, and São Paulo), that between Recife and São Paulo had a statistically significant pairwise PhiPT value of 0.132 (*p* = 0.031). The other two pairs had low and statistically insignificant PhiPT values: 0.006 between Campinas and Recife (*p* = 0.261), and 0.031 between Campinas and São Paulo (*p* = 0.132). Within the United Kingdom, the three geographic populations (Aberdeen, Leeds, and London) were genetically indistinguishable from each other with a PhiPT value of 0.000 for both between Aberdeen and Leeds (*p* = 0.398) and between Leeds and London (*p* = 0.352). The PhiPT value between Aberdeen and London was slightly higher than 0.000, at 0.017 and statistically also insignificant (*p* = 0.299).

The pairwise genetic differences among regional populations in China are presented in Table 6. Interestingly, different from those from the United Kingdom and to some extent from Brazil, most pairs of regional populations in China (22 of the 28 pairs) were statistically highly significantly differentiated. The only regional sample that showed no differentiation from the rest was that from Sichuan province. In contrast, pairs of populations from the remaining 7 regions were all significantly differentiated. The regional sample showing overall the biggest differences (PhiPT values ranging from 0.112 to 0.269) from other samples was that from Jiangxi province located in south central China, followed by that from Heilongjiang province in far northeastern China.

**Table 6:** Genetic differentiation between pairs of geographic samples of *C. tropicalis* from different regions in China.

	Beijing	Hainan	Heilongjiang	Jiangxi	Shanghai	Guangdong	Sichuan	Taiwan
Beijing		0.001	0.005	0.001	0.001	0.001	0.093	0.001
Hainan	0.034		0.001	0.001	0.001	0.013	0.392	0.001
Heilongjiang	0.117	0.125		0.001	0.005	0.015	0.109	0.001
Jiangxi	**0.112**	**0.200**	**0.248**		0.001	0.001	0.001	0.001
Shanghai	0.088	0.081	0.107	**0.221**		0.007	0.052	0.001
Guangdong	0.045	0.020	0.069	**0.238**	0.036		0.363	0.003
Sichuan	0.025	0.000	0.060	**0.269**	0.042	0.004		0.178
Taiwan	0.050	0.025	0.100	**0.196**	0.073	0.030	0.016	


*Only regions with more than 10 isolates were included in this analysis. PhiPT values are below diagonal. Probability, P(rand > = data) based on 999 permutations is shown above diagonal. Bolded numbers show the geographic population most distinct from other geographic populations.*

**FIGURE 1:**
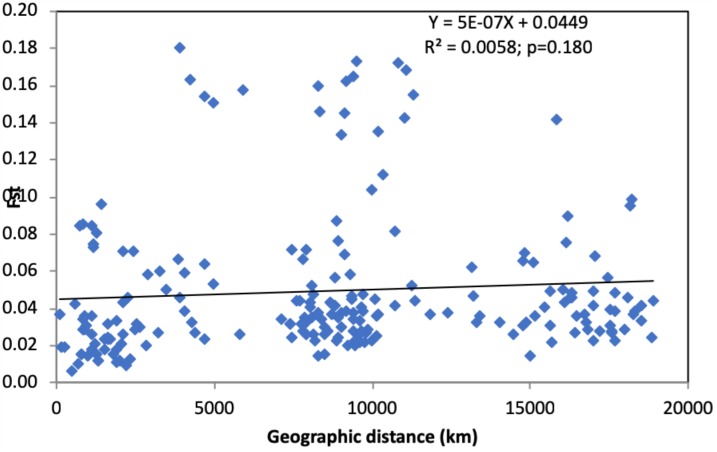
The relationship between genetic differentiation and geographic distance between pairs of regional and national samples of *Candida tropicalis*. Only regional or national samples with sample sizes greater than 10 were included in this analysis. *X*-axis: geographic distance, *Y*-axis: pairwise *F*_ST_ values. No statistically significant correlation was observed between geographic distance and genetic difference in the global sample of *C. tropicalis* (*p* = 0.180).

These results suggest that even though there is gene flow among geographic populations (also evidenced by multilocus genotype sharing across geographic scales), the amount of gene flow between most regional and national populations is insufficient to obscure the genetic differences among most of these populations. However, despite the observed significant genetic differentiation between most geographic populations, there is no evidence of genetic isolation by geographic distance (Figure 1).

### STRUCTURE Analyses

To identify potentially distinct genetic populations within the global *C. tropicalis* sample, we investigated the likely number of genetic clusters using the STRUCTURE software. Our analyses revealed an optimal number of 15 genetic clusters. The geographic distributions and sample sizes of these genetic clusters are presented in Table 7. The sample sizes of the genetic clusters ranged from 40 (Pop7) to 74 strains (Pop4), with each genetic cluster being relatively well represented in the global sample. Each of the 15 genetic clusters is distributed in at least two countries. Among these 15 genetic clusters, two (Pop6 and Pop14) are so far found only in Asia while each of the remaining 13 have been found in at least two continents. Three genetic clusters (Pop4, Pop7, and Pop12) have been found in four continents and two genetic clusters (Pop2 and Pop9) are found in all five continents from where multilocus sequences of *C. tropicalis* have been deposited in the MLST database.

**Table 7:** Distribution of the 15 inferred genetic clusters of *C. tropicalis* at the national level.

Genetic Clusters (no. of strains)	% prevalence (no. of strains) in indicated country																
	CHN	KOR	SGP	IND	BEL	DEU	GRC	ESP	SWE	United Kingdom	United States	AUS	NLD	ARG	BRA	COL.	UNK
Pop1 (72)	74 (53)	4 (3)	1 (1)	3 (2)	1 (1)			1 (1)		14 (10)					1 (1)		
Pop2 (60)	55 (33)	3 (2)		5 (3)						18 (11)	5 (3)	2 (1)			10 (6)	2 (1)	
Pop3 (55)	34 (19)				7 (4)	2 (1)	2 (1)		2 (1)	51 (28)	2 (1)						
Pop4 (74)	42 (31)	3 (2)		8 (6)	1 (1)					12 (9)	8 (6)		1 (1)		18 (13)	7 (5)	
Pop5 (55)	71 (39)									29 (16)							
Pop6 (53)	62 (33)		21 (11)	17 (9)													
Pop7 (40)	35 (14)				3 (1)					30 (12)	12 (5)			10 (4)	10 (4)		
Pop8 (47)	73 (34)	21 (10)											2 (1)		4 (2)		
Pop9 (63)	55 (35)	2 (1)		6 (4)						20 (13)	2 (1)	5 (3)			10 (6)		
Pop10 (53)	60 (32)			2 (1)						30 (16)					8 (4)		
Pop11 (63)	95 (60)	3 (2)													2 (1)		
Pop12 (48)	77 (37)		2 (1)		4 (2)					6 (3)	2 (1)				2 (1)	6 (3)	
Pop13 (73)	80 (58)				1 (1)	3 (2)				4 (3)			1 (1)		10 (7)		1 (1)
Pop14 (52)	98 (51)	2 (1)															
Pop15 (68)	93 (63)	1 (1)								4 (3)					1 (1)		


*CHN, China; KOR, South Korea; SGP, Singapore; IND, India; BEL, Belgium; DEU, Germany; GRC, Greece; ESP, Spain; SWE, Sweden; United Kingdom, United Kingdom; United States, United States of America; AUS, Australia; NLD, Netherlands; ARG, Argentina; BRA, Brazil; COL, Colombia; UNK, Unknown geographic origin.*

At the individual country-level, most countries with sample sizes greater than 10 contained strains belonging to at least two different genetic clusters. The two countries with the most strains in the database also had the highest numbers of genetic clusters. Specifically, all 15 genetic clusters are well-represented in China, the country with the highest number of strains in the database. Indeed, over 90% of the strains in three of the 15 genetic clusters (Pop11, Pop14, and Pop15) came from China. Similarly, 12 of the 15 genetic clusters are found in the United Kingdom sample. Taken together, the STRUCTURE analyses indicate that there are multiple distinct genetic clusters in the global sample of *C. tropicalis* and that most genetic clusters are broadly distributed, with some showing geographic bias. The results are overall consistent with the geographic analyses of multilocus genotype distributions, pairwise PhiPT, and AMOVA.

## Discussion

This study analyzed the geographic patterns of genotype variation in a global sample of *C. tropicalis*. Our analyses revealed that over 10% of the 2677 nucleotides at the six sequenced gene fragments were polymorphic in this global sample. These SNPs generated a large number of genotypes at each locus. In total, the DNA sequence variation at the six loci allowed the identification of 633 multilocus genotypes among the 876 strains. Among these genotypes, 93 were shared by 336 strains while the remaining 540 were represented by one strain each. The majority of the 93 shared multilocus genotypes were between strains from the same geographic region. However, strains from different regions within a country, from different countries, and from different continents have also been found to share identical multilocus genotypes. These results are consistent with short- medium-, and long- distance dispersals of *C. tropicalis* strains.

Population genetic analyses revealed that most genetic variations were found within regional and national samples. However, statistically significant genetic differences were found between many regional populations within China and between most national populations. Consistent with the high genetic diversity within most regional and national populations, STRUCTURE results showed that the global *C. tropicalis* sample belonged to 15 genetic clusters, with most genetic clusters distributed in multiple regions and countries. Overall, these results suggest that the global *C. tropicalis* population have likely been historically differentiated but recent gene flows have brought strains with divergent genotypes together into the same geographic regions. A similar result has been reported in another human fungal pathogen *Aspergillus fumigatus*. In that study, the analyses of genotype data at nine microsatellite loci for over 2000 strains revealed eight distinct genetic clusters with most clusters being broadly distributed in Asia, Europe, Australia, and South and North America (Ashu et al., 2017). Similar to that proposed for *A. fumigatus*, the dispersals observed here for *C. tropicalis* were likely due to anthropogenic factors such as commercial trade and human travel.

Our study relied on data from previous *C. tropicalis* MLST studies and the tremendous effort by the curator Dr. Frank Odds and his team in maintaining the database, to ensure the quality of submitted sequence data. For example, even though 742 DSTs were originally assigned by the end of May 2018 based on the initial sequence submissions by investigators, 109 of these DSTs were later invalidated based on critical inspections of submitted sequences (Table 2 and Supplementary Table 1). Due to the diploid nature of *C. tropicalis*, automatic base calls of sequence chromatographs can produce incorrect sequence reads and heterozygous sites may be mistakenly called homozygous. As a result, close inspection of sequence file is essential for ensuring the accuracy of the sequence information for each strain at each locus.

Previous studies of *C. tropicalis* MLST variation have mostly focused on local and regional samples and examined the potential relationships between strains with regard to patient characteristics (including age, sex, underlying condition, etc), source of infection, route of transmission, and the potential effects of antifungal drug treatments on genotypic changes within individual patients and hospital environments (e.g., Chen et al., 2009; Silva et al., 2012; Yang et al., 2012; Fan et al., 2014; Al-Obaid et al., 2017; Scordino et al., 2018). In general, these individual hospital-based or geographic region – specific studies revealed limited evidence for significant contribution of host body sites or other host factors to *C. tropicalis* genetic diversity in humans. For example, there were multiple cases of multilocus genotype sharing among unrelated individuals from different body sites and different geographic regions (e.g., Li et al., 2009; Wang et al., 2016; Supplementary Table 1). In one example, the most commonly shared genotype #140 was shared by strains from different anatomic sites among unrelated people in Taiwan and this result was hypothesized as evidence of a clonal expansion due to the strain’s ability to grow at high fluconazole concentration (Li et al., 2009). Our human anatomic site-based AMOVA of the global *C. tropicalis* samples identified that the anatomic sites in humans (blood, feces, oropharynx, other sterile body sites, skin and other superficial surfaces, urine, vagina) contributed only 0.5% to the total genetic variation (detailed data not shown). Together, the results suggest that at the global level, there is very little evidence for human anatomic site-specific adaptation of *C. tropicalis* MLST genotypes and that most genotypes and genetic clusters are capable of colonizing a diversity of human body sites and causing infections. We would like to emphasize that our current study focuses on the geographic patterns of *C. tropicalis* genetic variation based on the published and publicly available MLST data. Our study differs from previous studies by taking a broad population genetic approach to examine the distributions of genotypes and genetic variations across large geographic scales. While several broad and interesting patterns have emerged through our analyses, the amount of genetic diversity as well as the patterns of variation as revealed here are likely incomplete and biased. Below we discuss two areas of research that could help enhance our understanding of the global *C. tropicalis* population.

The first is to expand geographic sampling and representation of *C. tropicalis* in the MLST database. At present, the samples in the MLST database are geographically highly skewed at the regional, national, and continental levels. Less that 10% of the countries are represented in the database and literature. There are few or no isolates from most countries, including those in the entire African continent. Though the total number of *C. tropicalis* strains analyzed and deposited in the MLST database so far is large (876), over two-thirds of the strains were from one country, China. The second biggest source of the analyzed strains was the United Kingdom. However, over 50% (67/124) of the United Kingdom strains was from the first MLST study of *C. tropicalis* that established the consensus MLST loci and the database (Tavanti et al., 2005). Indeed, most or all strains in the *C. tropicalis* MLST database from several countries were deposited by this first *C. tropicalis* MLST study (Tavanti et al., 2005), including those from Australia (4 of 7 strains), United States (all 17 strains), Belgium (9 of 10 strains), Netherlands and Germany (3 strains from each of the two countries), and Greece and Sweden (1 strain from each country). It should be noted that the subsequent lack of data from some of these and many other developed countries was not due to the reduced clinical importance of *C. tropicalis* in these and other countries. In fact, *C. tropicalis* is often the second most common yeast species colonizing mucosal surfaces and causing bloodstream infections in many regions and countries (Capoor et al., 2005; Odds et al., 2007; Falagas et al., 2010; Adhikary and Joshi, 2011; Wang et al., 2013; Vaezi et al., 2017; Wu et al., 2017; Zuza-Alves et al., 2017). In addition, strains of *C. tropicalis* are often more resistant to the azole and polyene antifungal drugs than those of most other *Candida* species, including *C. albicans*, causing complications in disease treatments (Oliver et al., 2005; Chou et al., 2007; Desnos-Ollivier et al., 2008; Wu et al., 2014; Wang et al., 2016). Instead, we believe the skewed sample sizes among regions and countries in the *C. tropicalis* MLST database most likely reflect the efforts (or lack of) by medical mycologists from different countries and geographic regions over the last decade. As evidenced by a recent study in the journal *Mycopathologia*, studies of medical fungi have undergone a notable shift, with an increasing number of papers coming from emerging economies such as China, India, and Brazil etc. (Chaturvedi et al., 2018).

The second area of future research is to integrate natural ecological studies of *C. tropicalis* into clinical investigations and global population genetic analyses. *C. tropicalis* has been found in a diversity of ecological niches, including multiple anatomical sites in humans (Supplementary Table 1), soil (Yang et al., 2012; Aljohani et al., 2018), trees (Carvalho et al., 2014), aquatic environments (Vogel et al., 2007), and animals such as wild birds (Lord et al., 2010), horses (Cordeiro et al., 2013), rheas (Brilhante et al., 2013) as well as in tortoises and sea turtles (Brilhante et al., 2015). However, as seen in Supplementary Table 1, most geographic populations (at either the regional or national levels) in the current database had only a limited number of analyzed ecological niches (mostly in humans) and that most body sites had relatively few strains of *C. tropicalis* each. The small sample sizes in most geographic and ecological populations prevented us from conducting a robust analysis on the potential influences of ecological factors on the overall patterns of genetic variation. Even though there was a relatively large number of strains from Mainland China for most of the anatomic body sites (45 from blood, 11 from feces, 172 from the oral mucosal and oropharynx, four from sterile body sites other than blood, 32 from the skin and other superficial surfaces, 30 from urine, and 42 from vaginal tract), these samples were from geographically very diverse and distant provinces within China and most provincial populations only had one or two body sites with reasonable sample sizes (Supplementary Table 1). As a result, it’s difficult to estimate the potential contributions of ecological niches to the overall genetic variation without taking into account of the influences of geography at the same time. Though our preliminary analysis revealed that the global samples of *C. tropicalis* from different anatomic body sites had very little genetic difference from each other, it’s possible that populations of *C. tropicalis* from humans differ from those around their environments. Indeed, a previous study based on PCR fingerprinting revealed that the clinical and tree hollow populations of human pathogenic yeasts (including *C. tropicalis*) in Hamilton, ON, Canada were different (Carvalho et al., 2014). To accurately investigate the potential ecological contributions to *C. tropicalis* genetic variation and the relationships between environmental and human samples, future studies should focus on obtaining and analyzing diverse ecological samples from each geographic region and from many different regions and countries using the same MLST system.

## Conclusion

Our study revealed that the global population of *C. tropicalis* consists multiple genetic clusters with most of the clusters broadly distributed geographically. Our analyses showed that most observed genetic variations were found at local and regional levels and rejected the hypothesis that the global populations of *C. tropicalis* were structured based on geographic distances. Evidence for both medium- and long- distance dispersals was found. This study reaffirmed that the *C. tropicalis* MLST database provided a powerful platform not only for sharing strain and genotype information among investigators but also for helping us identifying broad epidemiological and population genetic patterns. Our analyses revealed several knowledge gaps from which future studies of *C. tropicalis* ecology, population genetics and molecular epidemiology could be focused on.

## Author Contributions

J-YW retrieved and analyzed the data. JX guided the data analyses and drafted the manuscript. D-YZ, FM, and YZ participated in data analyses. All authors read and approved the manuscript.

## Conflict of Interest Statement

The authors declare that the research was conducted in the absence of any commercial or financial relationships that could be construed as a potential conflict of interest.

## Footnotes

1 http://pubmlst.org/ctropicalis/

## Supplementary Material

FIGURE S1 Relationships among all 876 strains in the *C. tropicalis* MLST database as determined by their concatenated sequences at six gene fragments. For each strain, the strain name is followed by continent, country, and (when available) region (province/state/city). Scale bar represents the percent nucleotide base difference (0–12% on the scale bar) between strains, adjusted for diploids.

TABLE S1 Information about all 876 Candida tropicalis isolates analyzed in this study. Included in this table for each isolate are isolate name, geographic source, ecological niche, and multilocus genotype at six gene loci.
