# Supplementary material for: Analyses of the Global Multilocus Genotypes of the Human Pathogenic Yeast Candida tropicalis
Source: Front Microbiol. 2019 Apr 26;10:900. doi: 10.3389/fmicb.2019.00900 (PMC6497803; doi:10.3389/fmicb.2019.00900)
Supplement: FIGURE S1 — Relationships among all 876 strains in the C. tropicalis MLST database as determined by their concatenated sequences at six gene fragments. For each strain, the strain name is followed by continent, country, and (when available) region (province/state/city). Scale bar represents the percent nucleotide base difference (0–12% on the scale bar) between strains, adjusted for diploids. [file Data_Sheet_1.pdf]

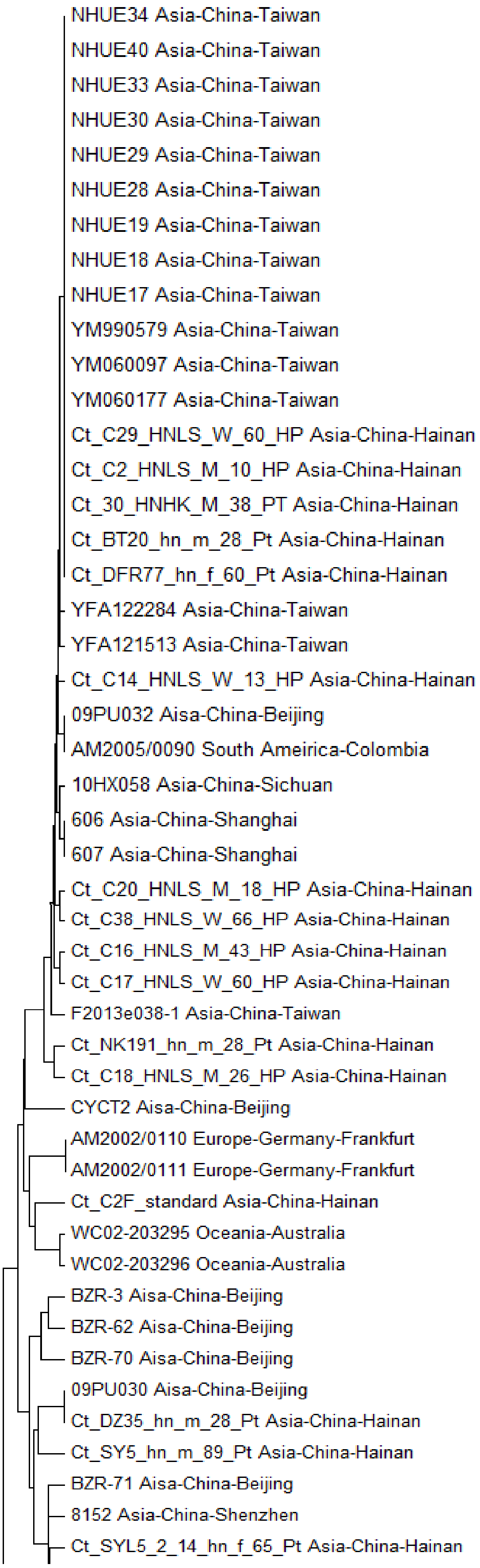
NHUE34 Asia-China-Taiwan
NHUE40 Asia-China-Taiwan
NHUE33 Asia-China-Taiwan
NHUE30 Asia-China-Taiwan
NHUE29 Asia-China-Taiwan
NHUE28 Asia-China-Taiwan
NHUE19 Asia-China-Taiwan
NHUE18 Asia-China-Taiwan
NHUE17 Asia-China-Taiwan
YM990579 Asia-China-Taiwan
YM060097 Asia-China-Taiwan
YM060177 Asia-China-Taiwan
Ct_C29_HNLS_W_60_HP Asia-China-Hainan
Ct_C2_HNLS_M_10_HP Asia-China-Hainan
Ct_30_HNHK_M_38_PT Asia-China-Hainan
Ct_BT20_hn_m_28_Pt Asia-China-Hainan
Ct_DFR77_hn_f_60_Pt Asia-China-Hainan
YFA122284 Asia-China-Taiwan
YFA121513 Asia-China-Taiwan
Ct_C14_HNLS_W_13_HP Asia-China-Hainan
09PU032 Aisa-China-Beijing
AM2005/0090 South Ameirica-Colombia
10HX058 Asia-China-Sichuan
606 Asia-China-Shanghai
607 Asia-China-Shanghai
Ct_C20_HNLS_M_18_HP Asia-China-Hainan
Ct_C38_HNLS_W_66_HP Asia-China-Hainan
Ct_C16_HNLS_M_43_HP Asia-China-Hainan
Ct_C17_HNLS_W_60_HP Asia-China-Hainan
F2013e038-1 Asia-China-Taiwan
Ct_NK191_hn_m_28_Pt Asia-China-Hainan
Ct_C18_HNLS_M_26_HP Asia-China-Hainan
CYCT2 Aisa-China-Beijing
AM2002/0110 Europe-Germany-Frankfurt
AM2002/0111 Europe-Germany-Frankfurt
Ct_C2F_standard Asia-China-Hainan
WC02-203295 Oceania-Australia
WC02-203296 Oceania-Australia
BZR-3 Aisa-China-Beijing
BZR-62 Aisa-China-Beijing
BZR-70 Aisa-China-Beijing
09PU030 Aisa-China-Beijing
Ct_DZ35_hn_m_28_Pt Asia-China-Hainan
Ct_SY5_hn_m_89_Pt Asia-China-Hainan
BZR-71 Aisa-China-Beijing
8152 Asia-China-Shenzhen
Ct_SYL5_2_14_hn_f_65_Pt Asia-China-Hainan

continued

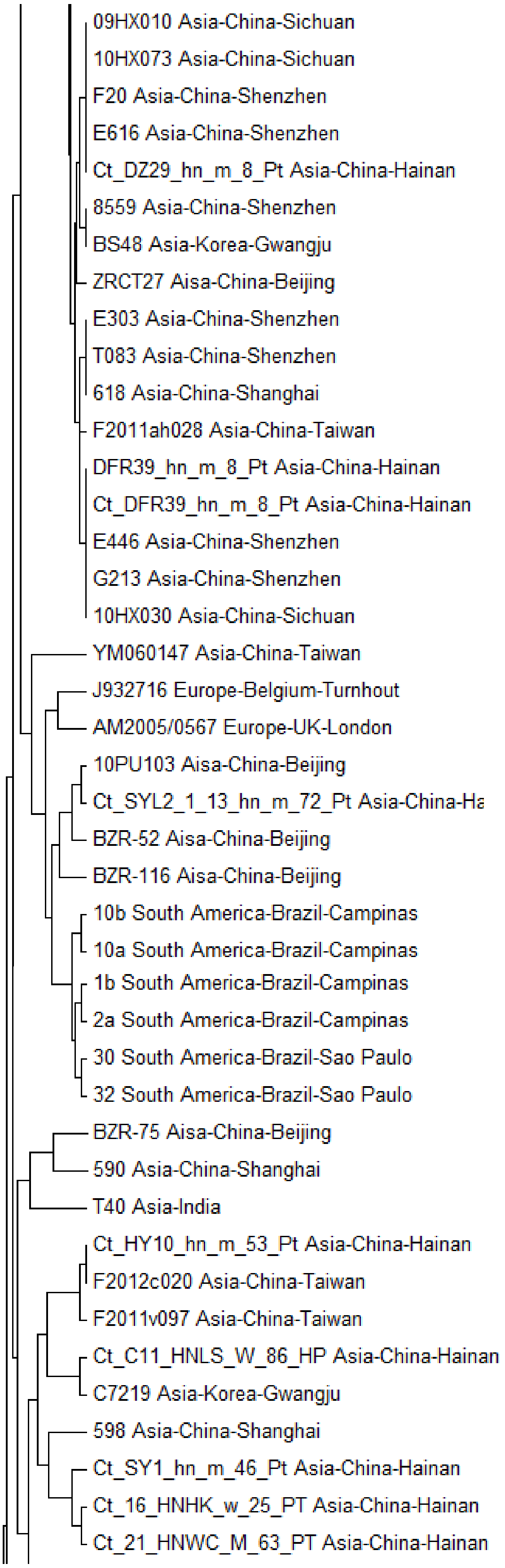
09HX010 Asia-China-Sichuan
10HX073 Asia-China-Sichuan
F20 Asia-China-Shenzhen
E616 Asia-China-Shenzhen
Ct_DZ29_hn_m_8_Pt Asia-China-Hainan
8559 Asia-China-Shenzhen
BS48 Asia-Korea-Gwangju
ZRCT27 Aisa-China-Beijing
E303 Asia-China-Shenzhen
T083 Asia-China-Shenzhen
618 Asia-China-Shanghai
F2011ah028 Asia-China-Taiwan
DFR39_hn_m_8_Pt Asia-China-Hainan
Ct_DFR39_hn_m_8_Pt Asia-China-Hainan
E446 Asia-China-Shenzhen
G213 Asia-China-Shenzhen
10HX030 Asia-China-Sichuan
YM060147 Asia-China-Taiwan
J932716 Europe-Belgium-Turnhout
AM2005/0567 Europe-UK-London
10PU103 Aisa-China-Beijing
Ct_SYL2_1_13_hn_m_72_Pt Asia-China-Ha
BZR-52 Aisa-China-Beijing
BZR-116 Aisa-China-Beijing
10b South America-Brazil-Campinas
10a South America-Brazil-Campinas
1b South America-Brazil-Campinas
2a South America-Brazil-Campinas
30 South America-Brazil-Sao Paulo
32 South America-Brazil-Sao Paulo
BZR-75 Aisa-China-Beijing
590 Asia-China-Shanghai
T40 Asia-India
Ct_HY10_hn_m_53_Pt Asia-China-Hainan
F2012c020 Asia-China-Taiwan
F2011v097 Asia-China-Taiwan
Ct_C11_HNLS_W_86_HP Asia-China-Hainan
C7219 Asia-Korea-Gwangju
598 Asia-China-Shanghai
Ct_SY1_hn_m_46_Pt Asia-China-Hainan
Ct_16_HNHK_w_25_PT Asia-China-Hainan
Ct_21_HNWC_M_63_PT Asia-China-Hainan

continued

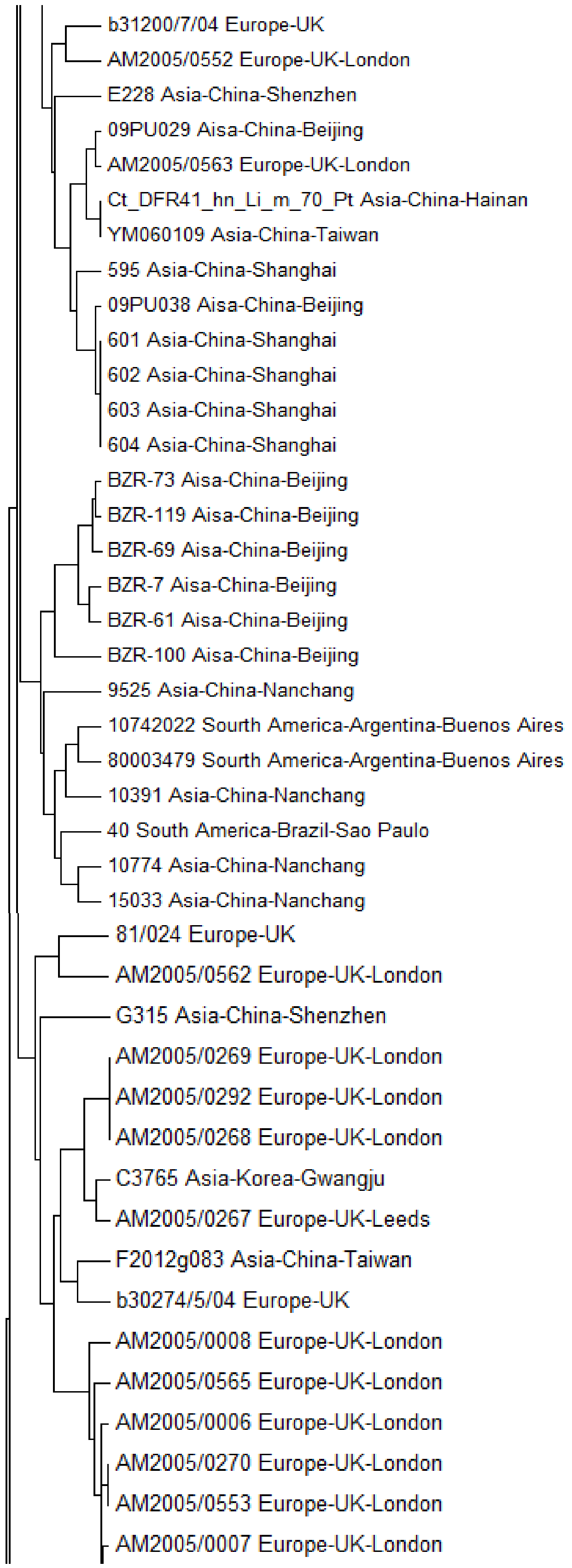
b31200/7/04 Europe-UK
AM2005/0552 Europe-UK-London
E228 Asia-China-Shenzhen
09PU029 Aisa-China-Beijing
AM2005/0563 Europe-UK-London
Ct_DFR41_hn_Li_m_70_Pt Asia-China-Hainan
YM060109 Asia-China-Taiwan
595 Asia-China-Shanghai
09PU038 Aisa-China-Beijing
601 Asia-China-Shanghai
602 Asia-China-Shanghai
603 Asia-China-Shanghai
604 Asia-China-Shanghai
BZR-73 Aisa-China-Beijing
BZR-119 Aisa-China-Beijing
BZR-69 Aisa-China-Beijing
BZR-7 Aisa-China-Beijing
BZR-61 Aisa-China-Beijing
BZR-100 Aisa-China-Beijing
9525 Asia-China-Nanchang
10742022 Sourth America-Argentina-Buenos Aires
80003479 Sourth America-Argentina-Buenos Aires
10391 Asia-China-Nanchang
40 South America-Brazil-Sao Paulo
10774 Asia-China-Nanchang
15033 Asia-China-Nanchang
81/024 Europe-UK
AM2005/0562 Europe-UK-London
G315 Asia-China-Shenzhen
AM2005/0269 Europe-UK-London
AM2005/0292 Europe-UK-London
AM2005/0268 Europe-UK-London
C3765 Asia-Korea-Gwangju
AM2005/0267 Europe-UK-Leeds
F2012g083 Asia-China-Taiwan
b30274/5/04 Europe-UK
AM2005/0008 Europe-UK-London
AM2005/0565 Europe-UK-London
AM2005/0006 Europe-UK-London
AM2005/0270 Europe-UK-London
AM2005/0553 Europe-UK-London
AM2005/0007 Europe-UK-London

continued

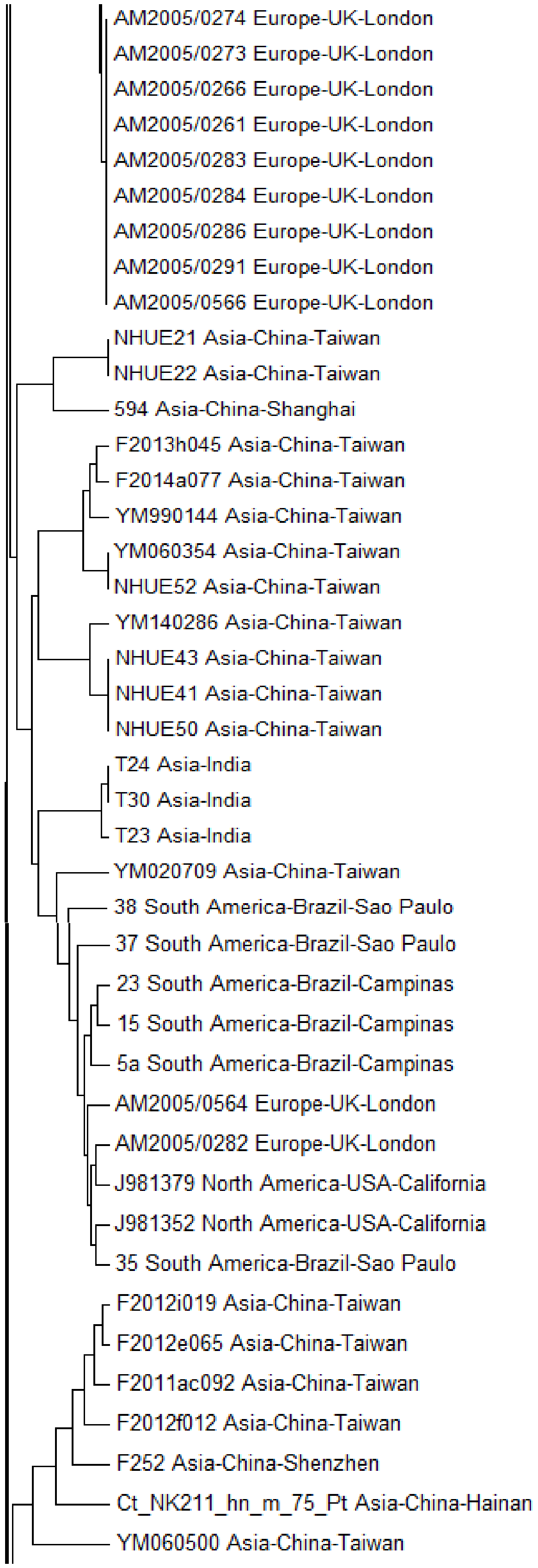
AM2005/0274 Europe-UK-London
AM2005/0273 Europe-UK-London
AM2005/0266 Europe-UK-London
AM2005/0261 Europe-UK-London
AM2005/0283 Europe-UK-London
AM2005/0284 Europe-UK-London
AM2005/0286 Europe-UK-London
AM2005/0291 Europe-UK-London
AM2005/0566 Europe-UK-London
NHUE21 Asia-China-Taiwan
NHUE22 Asia-China-Taiwan
594 Asia-China-Shanghai
F2013h045 Asia-China-Taiwan
F2014a077 Asia-China-Taiwan
YM990144 Asia-China-Taiwan
YM060354 Asia-China-Taiwan
NHUE52 Asia-China-Taiwan
YM140286 Asia-China-Taiwan
NHUE43 Asia-China-Taiwan
NHUE41 Asia-China-Taiwan
NHUE50 Asia-China-Taiwan
T24 Asia-India
T30 Asia-India
T23 Asia-India
YM020709 Asia-China-Taiwan
38 South America-Brazil-Sao Paulo
37 South America-Brazil-Sao Paulo
23 South America-Brazil-Campinas
15 South America-Brazil-Campinas
5a South America-Brazil-Campinas
AM2005/0564 Europe-UK-London
AM2005/0282 Europe-UK-London
J981379 North America-USA-California
J981352 North America-USA-California
35 South America-Brazil-Sao Paulo
F2012i019 Asia-China-Taiwan
F2012e065 Asia-China-Taiwan
F2011ac092 Asia-China-Taiwan
F2012f012 Asia-China-Taiwan
F252 Asia-China-Shenzhen
Ct_NK211_hn_m_75_Pt Asia-China-Hainan
YM060500 Asia-China-Taiwan

continued

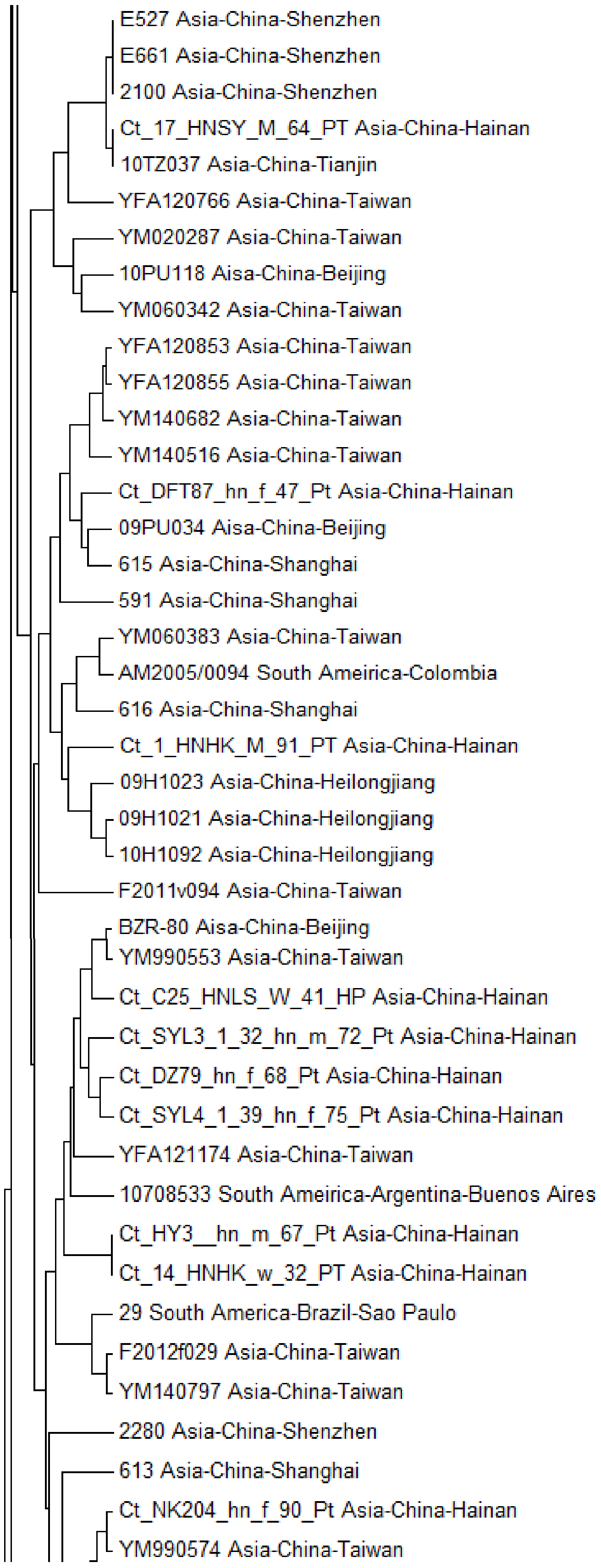
E527 Asia-China-Shenzhen
E661 Asia-China-Shenzhen
2100 Asia-China-Shenzhen
Ct_17_HNSY_M_64_PT Asia-China-Hainan
10TZ037 Asia-China-Tianjin
YFA120766 Asia-China-Taiwan
YM020287 Asia-China-Taiwan
10PU118 Aisa-China-Beijing
YM060342 Asia-China-Taiwan
YFA120853 Asia-China-Taiwan
YFA120855 Asia-China-Taiwan
YM140682 Asia-China-Taiwan
YM140516 Asia-China-Taiwan
Ct_DFT87_hn_f_47_Pt Asia-China-Hainan
09PU034 Aisa-China-Beijing
615 Asia-China-Shanghai
591 Asia-China-Shanghai
YM060383 Asia-China-Taiwan
AM2005/0094 South Ameirica-Colombia
616 Asia-China-Shanghai
Ct_1_HNHK_M_91_PT Asia-China-Hainan
09H1023 Asia-China-Heilongjiang
09H1021 Asia-China-Heilongjiang
10H1092 Asia-China-Heilongjiang
F2011v094 Asia-China-Taiwan
BZR-80 Aisa-China-Beijing
YM990553 Asia-China-Taiwan
Ct_C25_HNLS_W_41_HP Asia-China-Hainan
Ct_SYL3_1_32_hn_m_72_Pt Asia-China-Hainan
Ct_DZ79_hn_f_68_Pt Asia-China-Hainan
Ct_SYL4_1_39_hn_f_75_Pt Asia-China-Hainan
YFA121174 Asia-China-Taiwan
10708533 South Ameirica-Argentina-Buenos Aires
Ct_HY3__hn_m_67_Pt Asia-China-Hainan
Ct_14_HNHK_w_32_PT Asia-China-Hainan
29 South America-Brazil-Sao Paulo
F2012f029 Asia-China-Taiwan
YM140797 Asia-China-Taiwan
2280 Asia-China-Shenzhen
613 Asia-China-Shanghai
Ct_NK204_hn_f_90_Pt Asia-China-Hainan
YM990574 Asia-China-Taiwan

continued

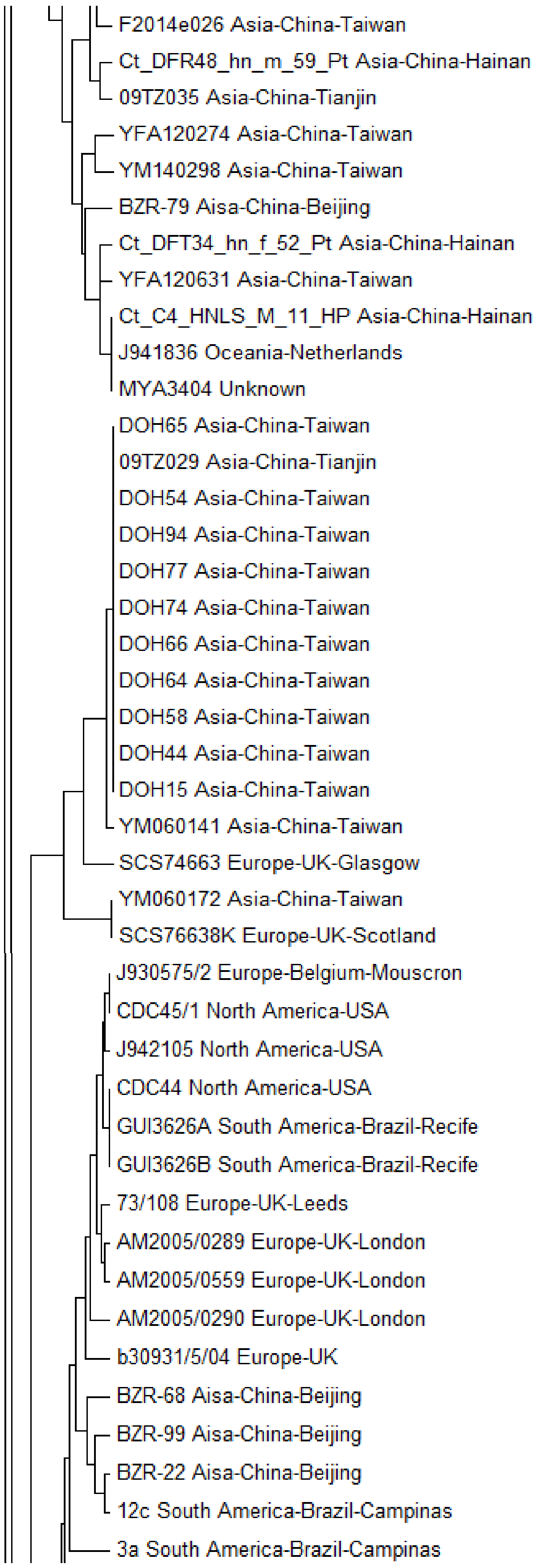
F2014e026 Asia-China-Taiwan
Ct_DFR48_hn_m_59_Pt Asia-China-Hainan
09TZ035 Asia-China-Tianjin
YFA120274 Asia-China-Taiwan
YM140298 Asia-China-Taiwan
BZR-79 Aisa-China-Beijing
Ct_DFT34_hn_f_52_Pt Asia-China-Hainan
YFA120631 Asia-China-Taiwan
Ct_C4_HNLS_M_11_HP Asia-China-Hainan
J941836 Oceania-Netherlands
MYA3404 Unknown
DOH65 Asia-China-Taiwan
09TZ029 Asia-China-Tianjin
DOH54 Asia-China-Taiwan
DOH94 Asia-China-Taiwan
DOH77 Asia-China-Taiwan
DOH74 Asia-China-Taiwan
DOH66 Asia-China-Taiwan
DOH64 Asia-China-Taiwan
DOH58 Asia-China-Taiwan
DOH44 Asia-China-Taiwan
DOH15 Asia-China-Taiwan
YM060141 Asia-China-Taiwan
SCS74663 Europe-UK-Glasgow
YM060172 Asia-China-Taiwan
SCS76638K Europe-UK-Scotland
J930575/2 Europe-Belgium-Mouscron
CDC45/1 North America-USA
J942105 North America-USA
CDC44 North America-USA
GUI3626A South America-Brazil-Recife
GUI3626B South America-Brazil-Recife
73/108 Europe-UK-Leeds
AM2005/0289 Europe-UK-London
AM2005/0559 Europe-UK-London
AM2005/0290 Europe-UK-London
b30931/5/04 Europe-UK
BZR-68 Aisa-China-Beijing
BZR-99 Aisa-China-Beijing
BZR-22 Aisa-China-Beijing
12c South America-Brazil-Campinas
3a South America-Brazil-Campinas

continued

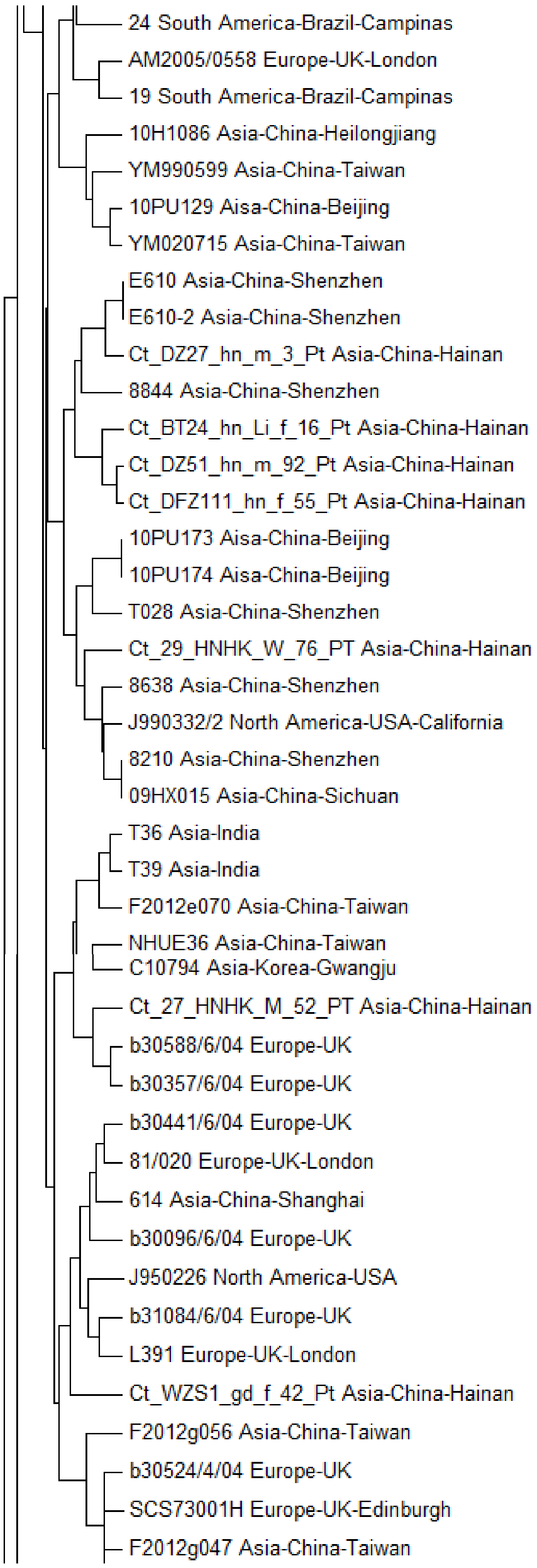
24 South America-Brazil-Campinas
AM2005/0558 Europe-UK-London
19 South America-Brazil-Campinas
10H1086 Asia-China-Heilongjiang
YM990599 Asia-China-Taiwan
10PU129 Aisa-China-Beijing
YM020715 Asia-China-Taiwan
E610 Asia-China-Shenzhen
E610-2 Asia-China-Shenzhen
Ct_DZ27_hn_m_3_Pt Asia-China-Hainan
8844 Asia-China-Shenzhen
Ct_BT24_hn_Li_f_16_Pt Asia-China-Hainan
Ct_DZ51_hn_m_92_Pt Asia-China-Hainan
Ct_DFZ111_hn_f_55_Pt Asia-China-Hainan
10PU173 Aisa-China-Beijing
10PU174 Aisa-China-Beijing
T028 Asia-China-Shenzhen
Ct_29_HNHK_W_76_PT Asia-China-Hainan
8638 Asia-China-Shenzhen
J990332/2 North America-USA-California
8210 Asia-China-Shenzhen
09HX015 Asia-China-Sichuan
T36 Asia-India
T39 Asia-India
F2012e070 Asia-China-Taiwan
NHUE36 Asia-China-Taiwan
C10794 Asia-Korea-Gwangju
Ct_27_HNHK_M_52_PT Asia-China-Hainan
b30588/6/04 Europe-UK
b30357/6/04 Europe-UK
b30441/6/04 Europe-UK
81/020 Europe-UK-London
614 Asia-China-Shanghai
b30096/6/04 Europe-UK
J950226 North America-USA
b31084/6/04 Europe-UK
L391 Europe-UK-London
Ct_WZS1_gd_f_42_Pt Asia-China-Hainan
F2012g056 Asia-China-Taiwan
b30524/4/04 Europe-UK
SCS73001H Europe-UK-Edinburgh
F2012g047 Asia-China-Taiwan

continued

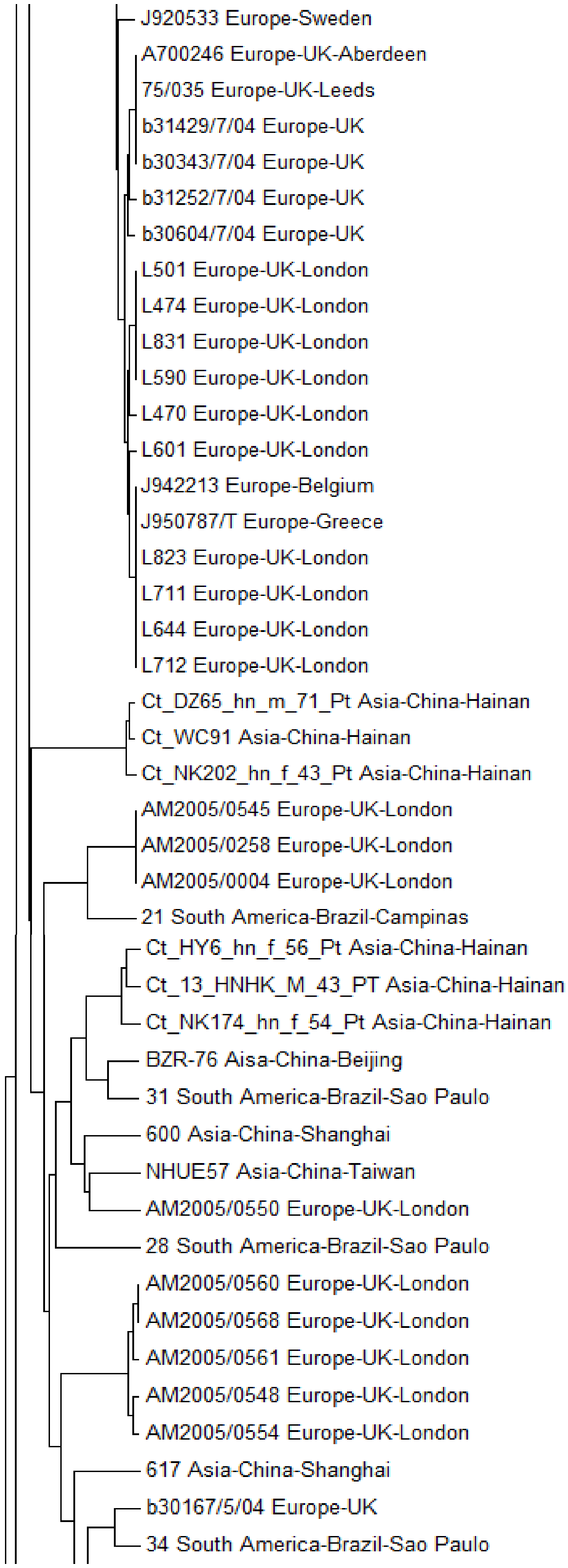
J920533 Europe-Sweden
A700246 Europe-UK-Aberdeen
75/035 Europe-UK-Leeds
b31429/7/04 Europe-UK
b30343/7/04 Europe-UK
b31252/7/04 Europe-UK
b30604/7/04 Europe-UK
L501 Europe-UK-London
L474 Europe-UK-London
L831 Europe-UK-London
L590 Europe-UK-London
L470 Europe-UK-London
L601 Europe-UK-London
J942213 Europe-Belgium
J950787/T Europe-Greece
L823 Europe-UK-London
L711 Europe-UK-London
L644 Europe-UK-London
L712 Europe-UK-London
Ct_DZ65_hn_m_71_Pt Asia-China-Hainan
Ct_WC91 Asia-China-Hainan
Ct_NK202_hn_f_43_Pt Asia-China-Hainan
AM2005/0545 Europe-UK-London
AM2005/0258 Europe-UK-London
AM2005/0004 Europe-UK-London
21 South America-Brazil-Campinas
Ct_HY6_hn_f_56_Pt Asia-China-Hainan
Ct_13_HNHK_M_43_PT Asia-China-Hainan
Ct_NK174_hn_f_54_Pt Asia-China-Hainan
BZR-76 Aisa-China-Beijing
31 South America-Brazil-Sao Paulo
600 Asia-China-Shanghai
NHUE57 Asia-China-Taiwan
AM2005/0550 Europe-UK-London
28 South America-Brazil-Sao Paulo
AM2005/0560 Europe-UK-London
AM2005/0568 Europe-UK-London
AM2005/0561 Europe-UK-London
AM2005/0548 Europe-UK-London
AM2005/0554 Europe-UK-London
617 Asia-China-Shanghai
b30167/5/04 Europe-UK
34 South America-Brazil-Sao Paulo

continued

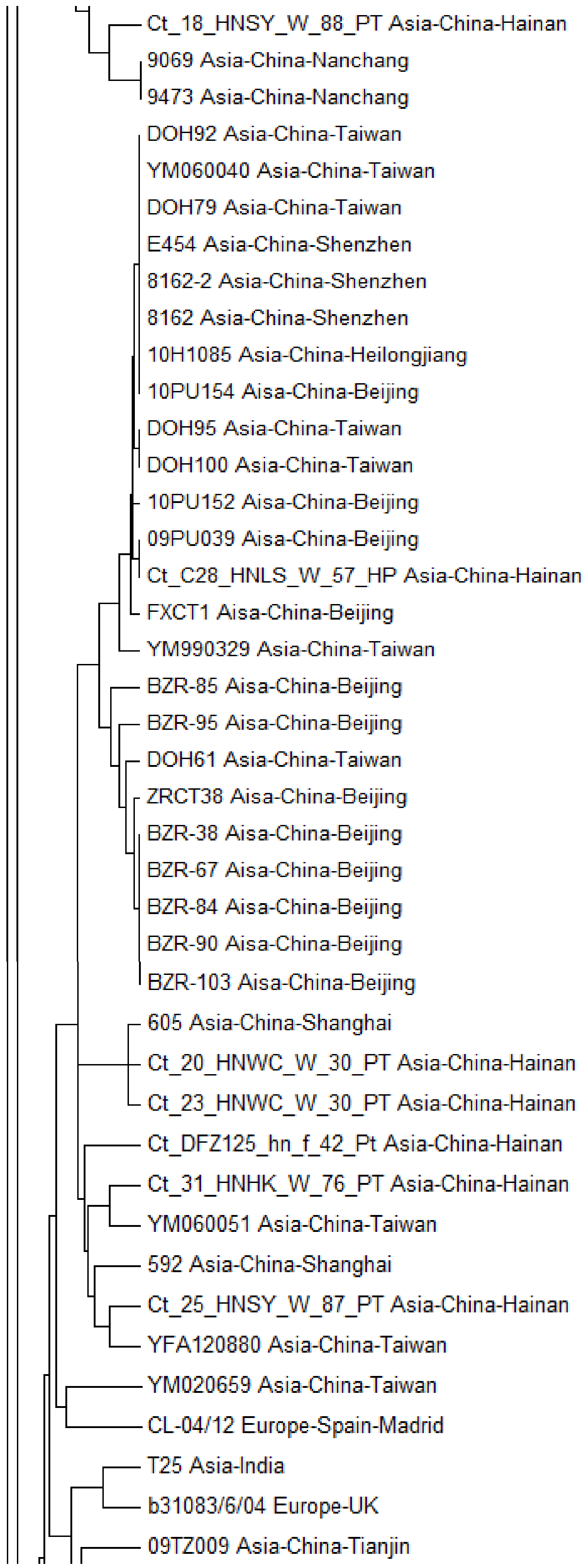
Ct_18_HNSY_W_88_PT Asia-China-Hainan
9069 Asia-China-Nanchang
9473 Asia-China-Nanchang
DOH92 Asia-China-Taiwan
YM060040 Asia-China-Taiwan
DOH79 Asia-China-Taiwan
E454 Asia-China-Shenzhen
8162-2 Asia-China-Shenzhen
8162 Asia-China-Shenzhen
10H1085 Asia-China-Heilongjiang
10PU154 Aisa-China-Beijing
DOH95 Asia-China-Taiwan
DOH100 Asia-China-Taiwan
10PU152 Aisa-China-Beijing
09PU039 Aisa-China-Beijing
Ct_C28_HNLS_W_57_HP Asia-China-Hainan
FXCT1 Aisa-China-Beijing
YM990329 Asia-China-Taiwan
BZR-85 Aisa-China-Beijing
BZR-95 Aisa-China-Beijing
DOH61 Asia-China-Taiwan
ZRCT38 Aisa-China-Beijing
BZR-38 Aisa-China-Beijing
BZR-67 Aisa-China-Beijing
BZR-84 Aisa-China-Beijing
BZR-90 Aisa-China-Beijing
BZR-103 Aisa-China-Beijing
605 Asia-China-Shanghai
Ct_20_HNWC_W_30_PT Asia-China-Hainan
Ct_23_HNWC_W_30_PT Asia-China-Hainan
Ct_DFZ125_hn_f_42_Pt Asia-China-Hainan
Ct_31_HNHK_W_76_PT Asia-China-Hainan
YM060051 Asia-China-Taiwan
592 Asia-China-Shanghai
Ct_25_HNSY_W_87_PT Asia-China-Hainan
YFA120880 Asia-China-Taiwan
YM020659 Asia-China-Taiwan
CL-04/12 Europe-Spain-Madrid
T25 Asia-India
b31083/6/04 Europe-UK
09TZ009 Asia-China-Tianjin

continued

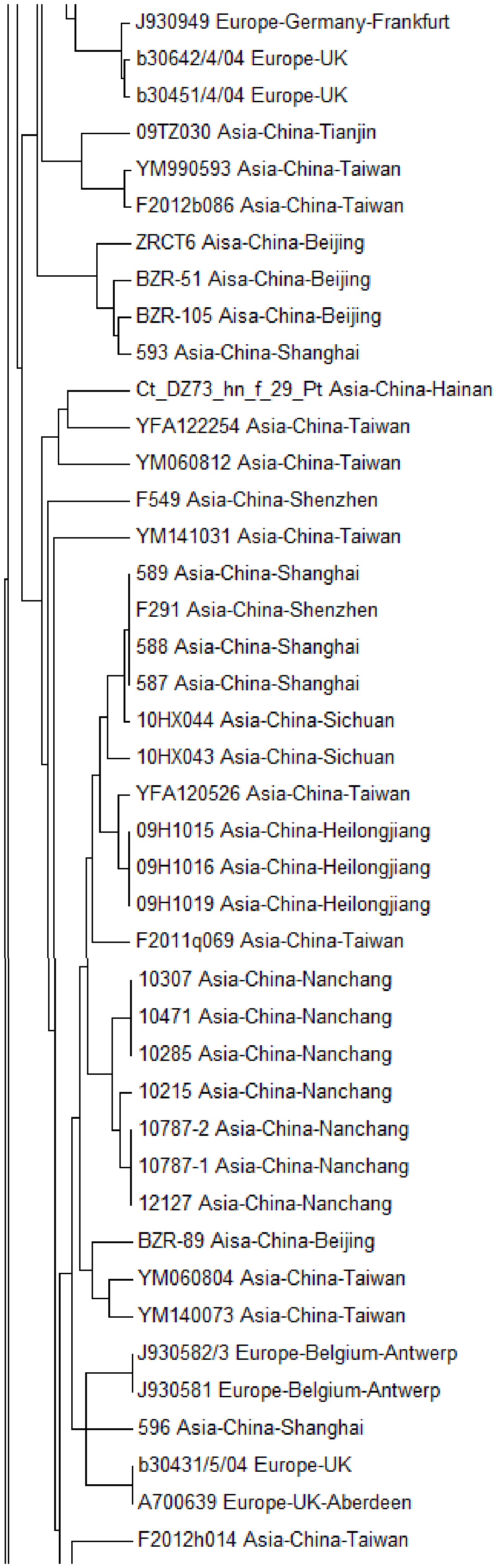
J930949 Europe-Germany-Frankfurt
b30642/4/04 Europe-UK
b30451/4/04 Europe-UK
09TZ030 Asia-China-Tianjin
YM990593 Asia-China-Taiwan
F2012b086 Asia-China-Taiwan
ZRCT6 Aisa-China-Beijing
BZR-51 Aisa-China-Beijing
BZR-105 Aisa-China-Beijing
593 Asia-China-Shanghai
Ct_DZ73_hn_f_29_Pt Asia-China-Hainan
YFA122254 Asia-China-Taiwan
YM060812 Asia-China-Taiwan
F549 Asia-China-Shenzhen
YM141031 Asia-China-Taiwan
589 Asia-China-Shanghai
F291 Asia-China-Shenzhen
588 Asia-China-Shanghai
587 Asia-China-Shanghai
10HX044 Asia-China-Sichuan
10HX043 Asia-China-Sichuan
YFA120526 Asia-China-Taiwan
09H1015 Asia-China-Heilongjiang
09H1016 Asia-China-Heilongjiang
09H1019 Asia-China-Heilongjiang
F2011q069 Asia-China-Taiwan
10307 Asia-China-Nanchang
10471 Asia-China-Nanchang
10285 Asia-China-Nanchang
10215 Asia-China-Nanchang
10787-2 Asia-China-Nanchang
10787-1 Asia-China-Nanchang
12127 Asia-China-Nanchang
BZR-89 Aisa-China-Beijing
YM060804 Asia-China-Taiwan
YM140073 Asia-China-Taiwan
J930582/3 Europe-Belgium-Antwerp
J930581 Europe-Belgium-Antwerp
596 Asia-China-Shanghai
b30431/5/04 Europe-UK
A700639 Europe-UK-Aberdeen
F2012h014 Asia-China-Taiwan

continued

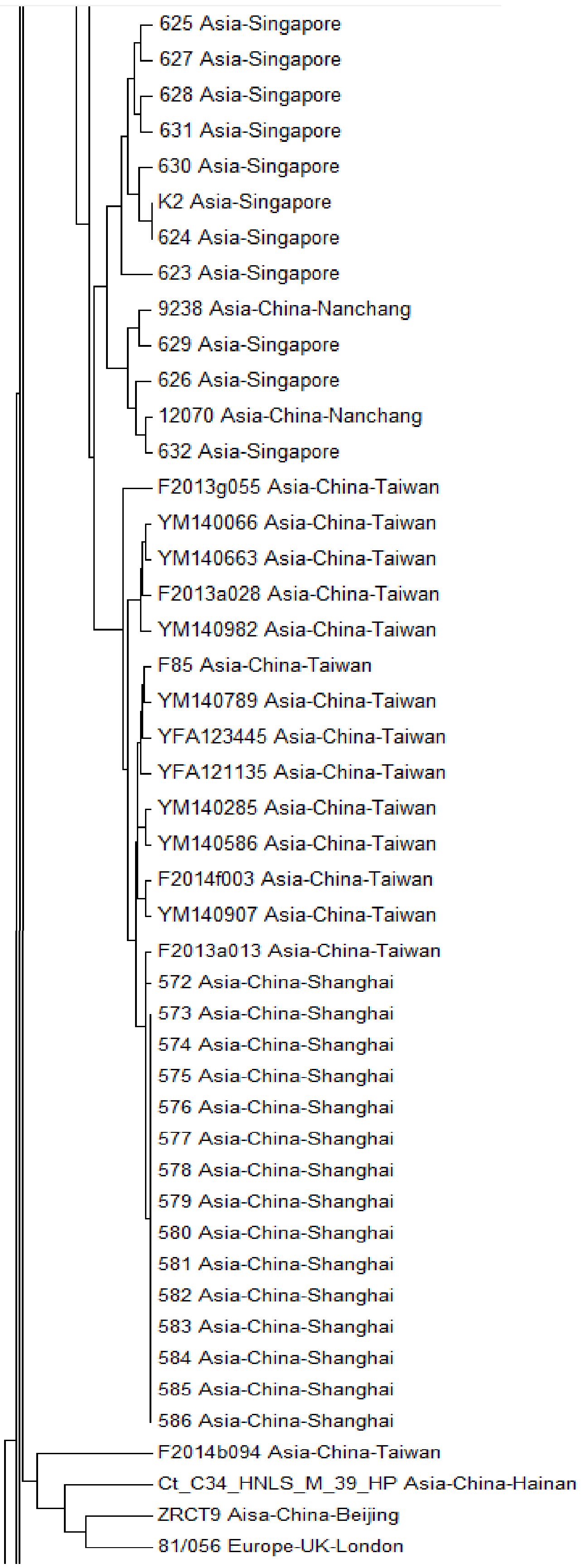
625 Asia-Singapore
627 Asia-Singapore
628 Asia-Singapore
631 Asia-Singapore
630 Asia-Singapore
K2 Asia-Singapore
624 Asia-Singapore
623 Asia-Singapore
9238 Asia-China-Nanchang
629 Asia-Singapore
626 Asia-Singapore
12070 Asia-China-Nanchang
632 Asia-Singapore
F2013g055 Asia-China-Taiwan
YM140066 Asia-China-Taiwan
YM140663 Asia-China-Taiwan
F2013a028 Asia-China-Taiwan
YM140982 Asia-China-Taiwan
F85 Asia-China-Taiwan
YM140789 Asia-China-Taiwan
YFA123445 Asia-China-Taiwan
YFA121135 Asia-China-Taiwan
YM140285 Asia-China-Taiwan
YM140586 Asia-China-Taiwan
F2014f003 Asia-China-Taiwan
YM140907 Asia-China-Taiwan
F2013a013 Asia-China-Taiwan
572 Asia-China-Shanghai
573 Asia-China-Shanghai
574 Asia-China-Shanghai
575 Asia-China-Shanghai
576 Asia-China-Shanghai
577 Asia-China-Shanghai
578 Asia-China-Shanghai
579 Asia-China-Shanghai
580 Asia-China-Shanghai
581 Asia-China-Shanghai
582 Asia-China-Shanghai
583 Asia-China-Shanghai
584 Asia-China-Shanghai
585 Asia-China-Shanghai
586 Asia-China-Shanghai
F2014b094 Asia-China-Taiwan
Ct_C34_HNLS_M_39_HP Asia-China-Hainan
ZRCT9 Aisa-China-Beijing
81/056 Europe-UK-London

continued

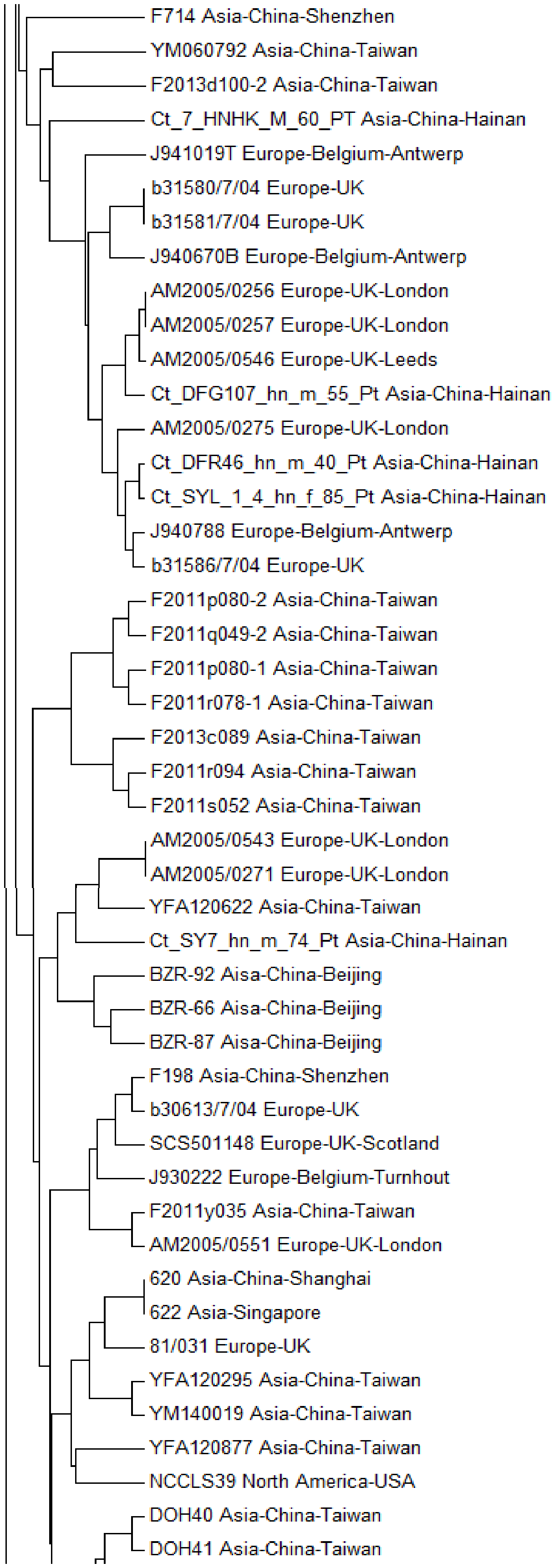
F714 Asia-China-Shenzhen
YM060792 Asia-China-Taiwan
F2013d100-2 Asia-China-Taiwan
Ct_7_HNHK_M_60_PT Asia-China-Hainan
J941019T Europe-Belgium-Antwerp
b31580/7/04 Europe-UK
b31581/7/04 Europe-UK
J940670B Europe-Belgium-Antwerp
AM2005/0256 Europe-UK-London
AM2005/0257 Europe-UK-London
AM2005/0546 Europe-UK-Leeds
Ct_DFG107_hn_m_55_Pt Asia-China-Hainan
AM2005/0275 Europe-UK-London
Ct_DFR46_hn_m_40_Pt Asia-China-Hainan
Ct_SYL_1_4_hn_f_85_Pt Asia-China-Hainan
J940788 Europe-Belgium-Antwerp
b31586/7/04 Europe-UK
F2011p080-2 Asia-China-Taiwan
F2011q049-2 Asia-China-Taiwan
F2011p080-1 Asia-China-Taiwan
F2011r078-1 Asia-China-Taiwan
F2013c089 Asia-China-Taiwan
F2011r094 Asia-China-Taiwan
F2011s052 Asia-China-Taiwan
AM2005/0543 Europe-UK-London
AM2005/0271 Europe-UK-London
YFA120622 Asia-China-Taiwan
Ct_SY7_hn_m_74_Pt Asia-China-Hainan
BZR-92 Aisa-China-Beijing
BZR-66 Aisa-China-Beijing
BZR-87 Aisa-China-Beijing
F198 Asia-China-Shenzhen
b30613/7/04 Europe-UK
SCS501148 Europe-UK-Scotland
J930222 Europe-Belgium-Turnhout
F2011y035 Asia-China-Taiwan
AM2005/0551 Europe-UK-London
620 Asia-China-Shanghai
622 Asia-Singapore
81/031 Europe-UK
YFA120295 Asia-China-Taiwan
YM140019 Asia-China-Taiwan
YFA120877 Asia-China-Taiwan
NCCLS39 North America-USA
DOH40 Asia-China-Taiwan
DOH41 Asia-China-Taiwan

continued

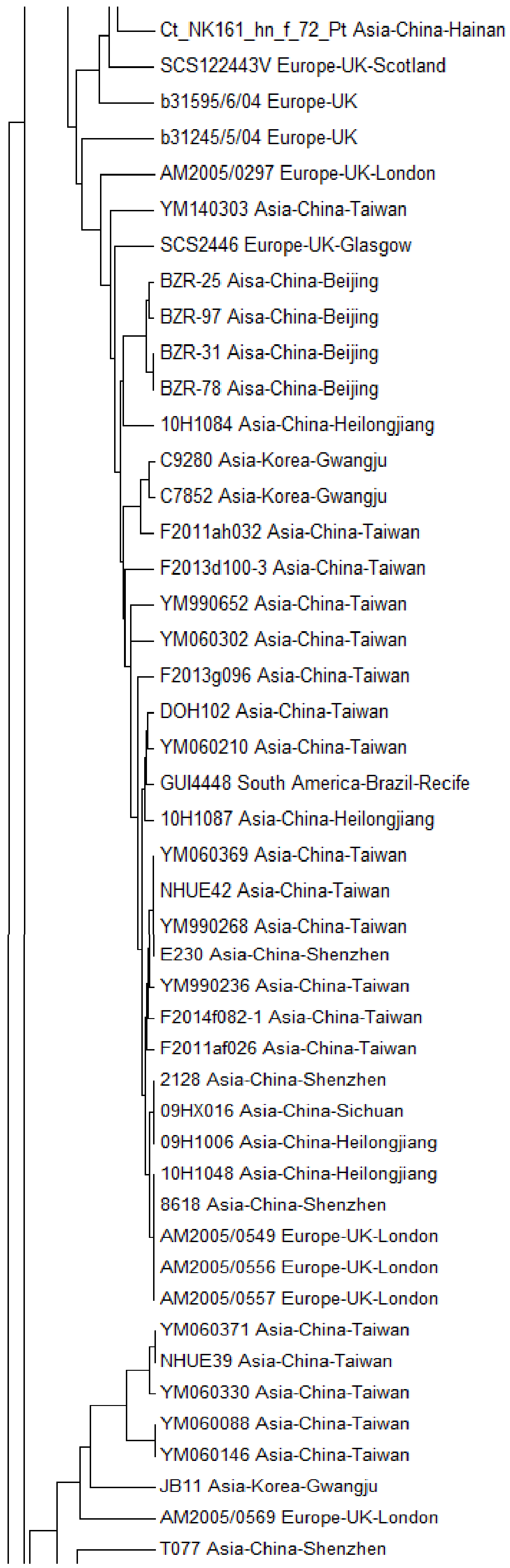
Ct_NK161_hn_f_72_Pt Asia-China-Hainan
SCS122443V Europe-UK-Scotland
b31595/6/04 Europe-UK
b31245/5/04 Europe-UK
AM2005/0297 Europe-UK-London
YM140303 Asia-China-Taiwan
SCS2446 Europe-UK-Glasgow
BZR-25 Aisa-China-Beijing
BZR-97 Aisa-China-Beijing
BZR-31 Aisa-China-Beijing
BZR-78 Aisa-China-Beijing
10H1084 Asia-China-Heilongjiang
C9280 Asia-Korea-Gwangju
C7852 Asia-Korea-Gwangju
F2011ah032 Asia-China-Taiwan
F2013d100-3 Asia-China-Taiwan
YM990652 Asia-China-Taiwan
YM060302 Asia-China-Taiwan
F2013g096 Asia-China-Taiwan
DOH102 Asia-China-Taiwan
YM060210 Asia-China-Taiwan
GUI4448 South America-Brazil-Recife
10H1087 Asia-China-Heilongjiang
YM060369 Asia-China-Taiwan
NHUE42 Asia-China-Taiwan
YM990268 Asia-China-Taiwan
E230 Asia-China-Shenzhen
YM990236 Asia-China-Taiwan
F2014f082-1 Asia-China-Taiwan
F2011af026 Asia-China-Taiwan
2128 Asia-China-Shenzhen
09HX016 Asia-China-Sichuan
09H1006 Asia-China-Heilongjiang
10H1048 Asia-China-Heilongjiang
8618 Asia-China-Shenzhen
AM2005/0549 Europe-UK-London
AM2005/0556 Europe-UK-London
AM2005/0557 Europe-UK-London
YM060371 Asia-China-Taiwan
NHUE39 Asia-China-Taiwan
YM060330 Asia-China-Taiwan
YM060088 Asia-China-Taiwan
YM060146 Asia-China-Taiwan
JB11 Asia-Korea-Gwangju
AM2005/0569 Europe-UK-London
T077 Asia-China-Shenzhen

continued

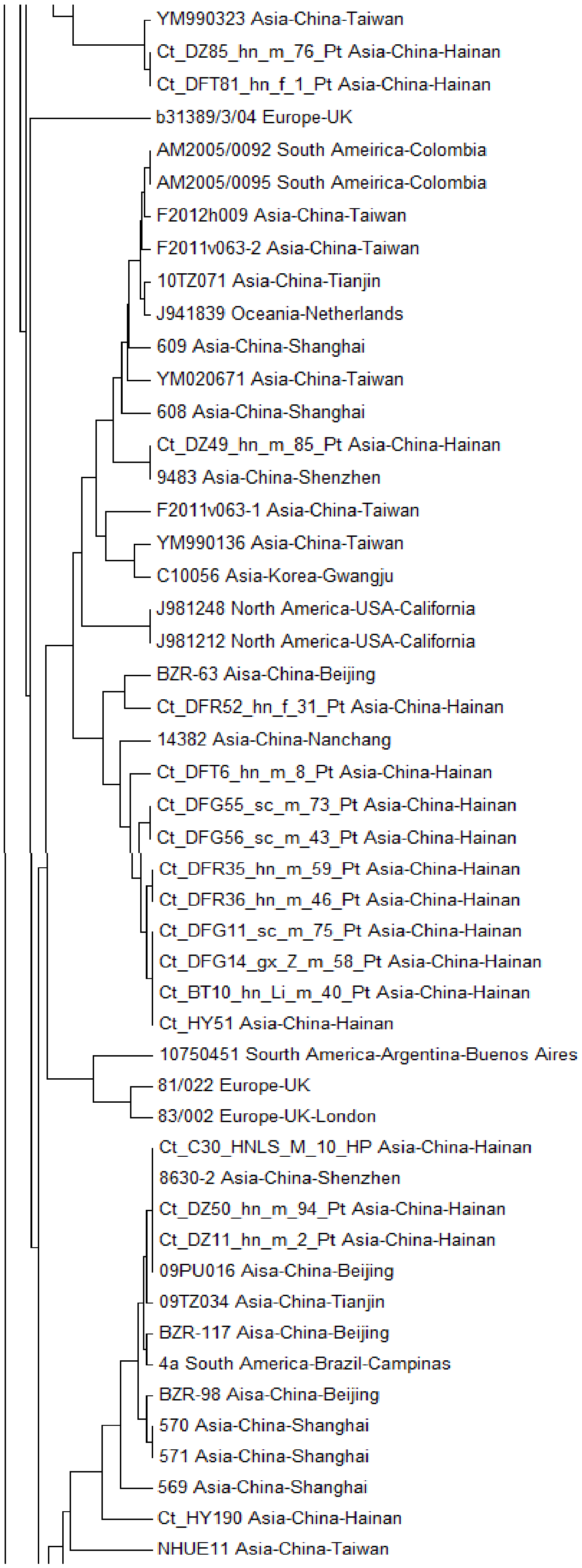
YM990323 Asia-China-Taiwan
Ct_DZ85_hn_m_76_Pt Asia-China-Hainan
Ct_DFT81_hn_f_1_Pt Asia-China-Hainan
b31389/3/04 Europe-UK
AM2005/0092 South Ameirica-Colombia
AM2005/0095 South Ameirica-Colombia
F2012h009 Asia-China-Taiwan
F2011v063-2 Asia-China-Taiwan
10TZ071 Asia-China-Tianjin
J941839 Oceania-Netherlands
609 Asia-China-Shanghai
YM020671 Asia-China-Taiwan
608 Asia-China-Shanghai
Ct_DZ49_hn_m_85_Pt Asia-China-Hainan
9483 Asia-China-Shenzhen
F2011v063-1 Asia-China-Taiwan
YM990136 Asia-China-Taiwan
C10056 Asia-Korea-Gwangju
J981248 North America-USA-California
J981212 North America-USA-California
BZR-63 Aisa-China-Beijing
Ct_DFR52_hn_f_31_Pt Asia-China-Hainan
14382 Asia-China-Nanchang
Ct_DFT6_hn_m_8_Pt Asia-China-Hainan
Ct_DFG55_sc_m_73_Pt Asia-China-Hainan
Ct_DFG56_sc_m_43_Pt Asia-China-Hainan
Ct_DFR35_hn_m_59_Pt Asia-China-Hainan
Ct_DFR36_hn_m_46_Pt Asia-China-Hainan
Ct_DFG11_sc_m_75_Pt Asia-China-Hainan
Ct_DFG14_gx_Z_m_58_Pt Asia-China-Hainan
Ct_BT10_hn_Li_m_40_Pt Asia-China-Hainan
Ct_HY51 Asia-China-Hainan
10750451 Sourth America-Argentina-Buenos Aires
81/022 Europe-UK
83/002 Europe-UK-London
Ct_C30_HNLS_M_10_HP Asia-China-Hainan
8630-2 Asia-China-Shenzhen
Ct_DZ50_hn_m_94_Pt Asia-China-Hainan
Ct_DZ11_hn_m_2_Pt Asia-China-Hainan
09PU016 Aisa-China-Beijing
09TZ034 Asia-China-Tianjin
BZR-117 Aisa-China-Beijing
4a South America-Brazil-Campinas
BZR-98 Aisa-China-Beijing
570 Asia-China-Shanghai
571 Asia-China-Shanghai
569 Asia-China-Shanghai
Ct_HY190 Asia-China-Hainan
NHUE11 Asia-China-Taiwan

continued

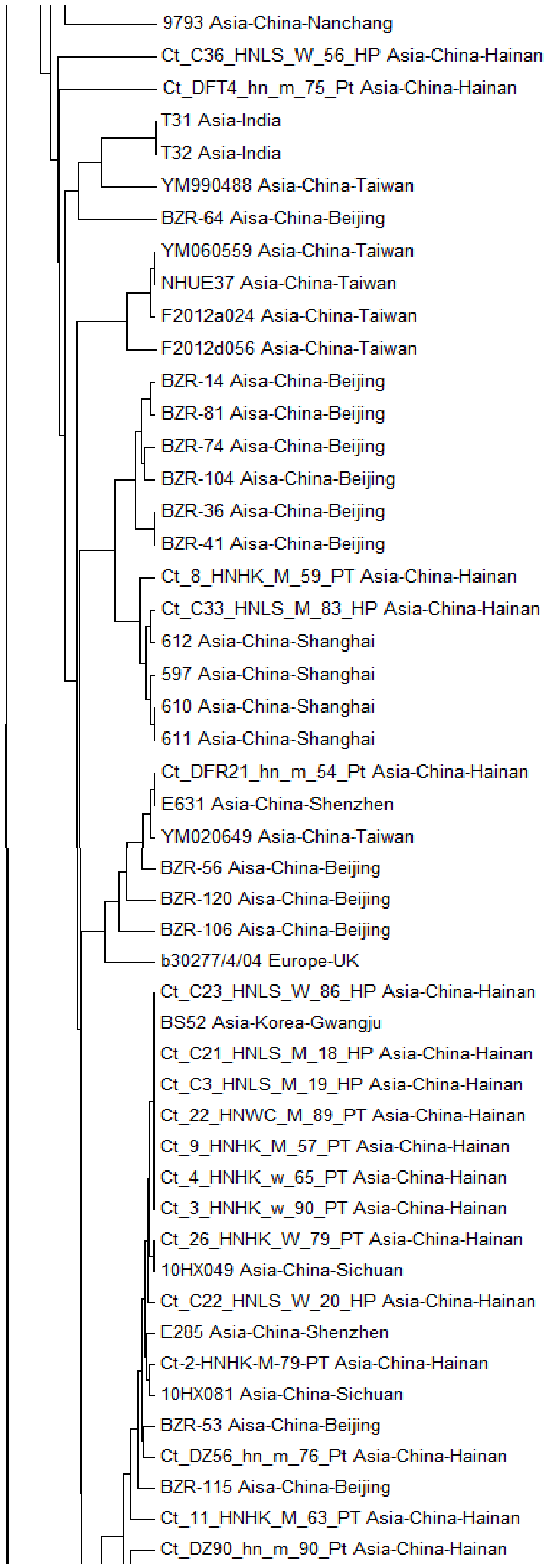
9793 Asia-China-Nanchang
Ct_C36_HNLS_W_56_HP Asia-China-Hainan
Ct_DFT4_hn_m_75_Pt Asia-China-Hainan
T31 Asia-India
T32 Asia-India
YM990488 Asia-China-Taiwan
BZR-64 Aisa-China-Beijing
YM060559 Asia-China-Taiwan
NHUE37 Asia-China-Taiwan
F2012a024 Asia-China-Taiwan
F2012d056 Asia-China-Taiwan
BZR-14 Aisa-China-Beijing
BZR-81 Aisa-China-Beijing
BZR-74 Aisa-China-Beijing
BZR-104 Aisa-China-Beijing
BZR-36 Aisa-China-Beijing
BZR-41 Aisa-China-Beijing
Ct_8_HNHK_M_59_PT Asia-China-Hainan
Ct_C33_HNLS_M_83_HP Asia-China-Hainan
612 Asia-China-Shanghai
597 Asia-China-Shanghai
610 Asia-China-Shanghai
611 Asia-China-Shanghai
Ct_DFR21_hn_m_54_Pt Asia-China-Hainan
E631 Asia-China-Shenzhen
YM020649 Asia-China-Taiwan
BZR-56 Aisa-China-Beijing
BZR-120 Aisa-China-Beijing
BZR-106 Aisa-China-Beijing
b30277/4/04 Europe-UK
Ct_C23_HNLS_W_86_HP Asia-China-Hainan
BS52 Asia-Korea-Gwangju
Ct_C21_HNLS_M_18_HP Asia-China-Hainan
Ct_C3_HNLS_M_19_HP Asia-China-Hainan
Ct_22_HNWC_M_89_PT Asia-China-Hainan
Ct_9_HNHK_M_57_PT Asia-China-Hainan
Ct_4_HNHK_w_65_PT Asia-China-Hainan
Ct_3_HNHK_w_90_PT Asia-China-Hainan
Ct_26_HNHK_W_79_PT Asia-China-Hainan
10HX049 Asia-China-Sichuan
Ct_C22_HNLS_W_20_HP Asia-China-Hainan
E285 Asia-China-Shenzhen
Ct-2-HNHK-M-79-PT Asia-China-Hainan
10HX081 Asia-China-Sichuan
BZR-53 Aisa-China-Beijing
Ct_DZ56_hn_m_76_Pt Asia-China-Hainan
BZR-115 Aisa-China-Beijing
Ct_11_HNHK_M_63_PT Asia-China-Hainan
Ct_DZ90_hn_m_90_Pt Asia-China-Hainan

continued

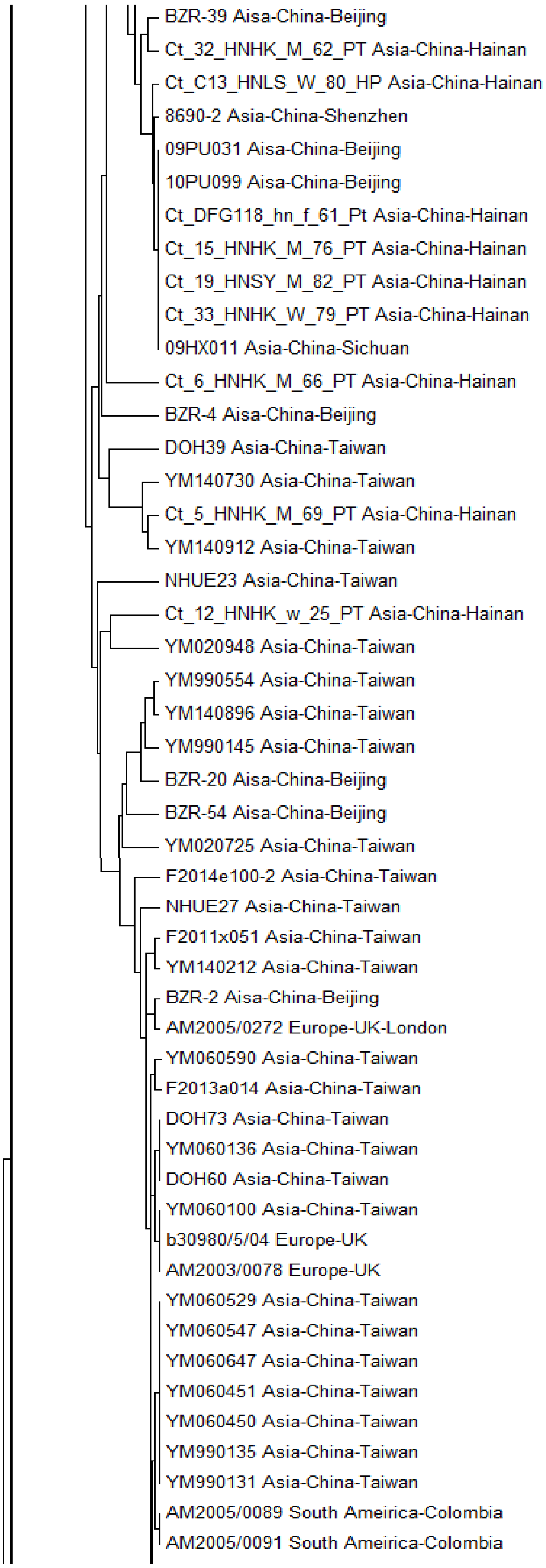
BZR-39 Aisa-China-Beijing
Ct_32_HNHK_M_62_PT Asia-China-Hainan
Ct_C13_HNLS_W_80_HP Asia-China-Hainan
8690-2 Asia-China-Shenzhen
09PU031 Aisa-China-Beijing
10PU099 Aisa-China-Beijing
Ct_DFG118_hn_f_61_Pt Asia-China-Hainan
Ct_15_HNHK_M_76_PT Asia-China-Hainan
Ct_19_HNSY_M_82_PT Asia-China-Hainan
Ct_33_HNHK_W_79_PT Asia-China-Hainan
09HX011 Asia-China-Sichuan
Ct_6_HNHK_M_66_PT Asia-China-Hainan
BZR-4 Aisa-China-Beijing
DOH39 Asia-China-Taiwan
YM140730 Asia-China-Taiwan
Ct_5_HNHK_M_69_PT Asia-China-Hainan
YM140912 Asia-China-Taiwan
NHUE23 Asia-China-Taiwan
Ct_12_HNHK_w_25_PT Asia-China-Hainan
YM020948 Asia-China-Taiwan
YM990554 Asia-China-Taiwan
YM140896 Asia-China-Taiwan
YM990145 Asia-China-Taiwan
BZR-20 Aisa-China-Beijing
BZR-54 Aisa-China-Beijing
YM020725 Asia-China-Taiwan
F2014e100-2 Asia-China-Taiwan
NHUE27 Asia-China-Taiwan
F2011x051 Asia-China-Taiwan
YM140212 Asia-China-Taiwan
BZR-2 Aisa-China-Beijing
AM2005/0272 Europe-UK-London
YM060590 Asia-China-Taiwan
F2013a014 Asia-China-Taiwan
DOH73 Asia-China-Taiwan
YM060136 Asia-China-Taiwan
DOH60 Asia-China-Taiwan
YM060100 Asia-China-Taiwan
b30980/5/04 Europe-UK
AM2003/0078 Europe-UK
YM060529 Asia-China-Taiwan
YM060547 Asia-China-Taiwan
YM060647 Asia-China-Taiwan
YM060451 Asia-China-Taiwan
YM060450 Asia-China-Taiwan
YM990135 Asia-China-Taiwan
YM990131 Asia-China-Taiwan
AM2005/0089 South Ameirica-Colombia
AM2005/0091 South Ameirica-Colombia

continued

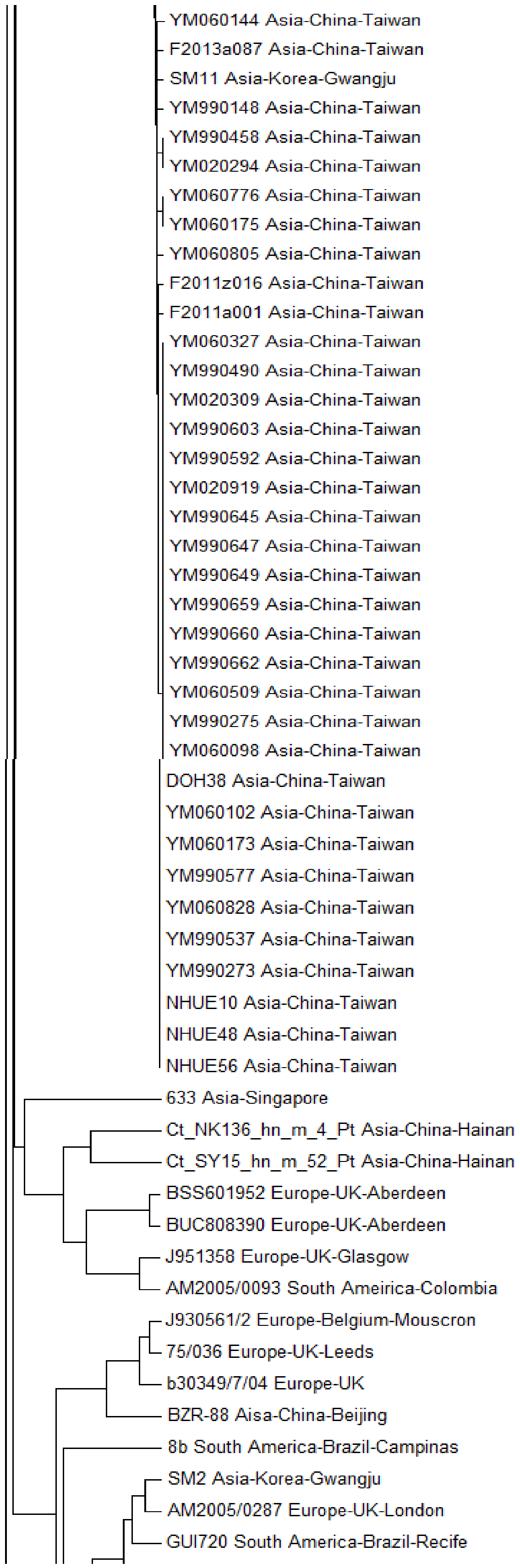
YM060144 Asia-China-Taiwan
F2013a087 Asia-China-Taiwan
SM11 Asia-Korea-Gwangju
YM990148 Asia-China-Taiwan
YM990458 Asia-China-Taiwan
YM020294 Asia-China-Taiwan
YM060776 Asia-China-Taiwan
YM060175 Asia-China-Taiwan
YM060805 Asia-China-Taiwan
F2011z016 Asia-China-Taiwan
F2011a001 Asia-China-Taiwan
YM060327 Asia-China-Taiwan
YM990490 Asia-China-Taiwan
YM020309 Asia-China-Taiwan
YM990603 Asia-China-Taiwan
YM990592 Asia-China-Taiwan
YM020919 Asia-China-Taiwan
YM990645 Asia-China-Taiwan
YM990647 Asia-China-Taiwan
YM990649 Asia-China-Taiwan
YM990659 Asia-China-Taiwan
YM990660 Asia-China-Taiwan
YM990662 Asia-China-Taiwan
YM060509 Asia-China-Taiwan
YM990275 Asia-China-Taiwan
YM060098 Asia-China-Taiwan
DOH38 Asia-China-Taiwan
YM060102 Asia-China-Taiwan
YM060173 Asia-China-Taiwan
YM990577 Asia-China-Taiwan
YM060828 Asia-China-Taiwan
YM990537 Asia-China-Taiwan
YM990273 Asia-China-Taiwan
NHUE10 Asia-China-Taiwan
NHUE48 Asia-China-Taiwan
NHUE56 Asia-China-Taiwan
633 Asia-Singapore
Ct_NK136_hn_m_4_Pt Asia-China-Hainan
Ct_SY15_hn_m_52_Pt Asia-China-Hainan
BSS601952 Europe-UK-Aberdeen
BUC808390 Europe-UK-Aberdeen
J951358 Europe-UK-Glasgow
AM2005/0093 South Ameirica-Colombia
J930561/2 Europe-Belgium-Mouscron
75/036 Europe-UK-Leeds
b30349/7/04 Europe-UK
BZR-88 Aisa-China-Beijing
8b South America-Brazil-Campinas
SM2 Asia-Korea-Gwangju
AM2005/0287 Europe-UK-London
GUI720 South America-Brazil-Recife

continued

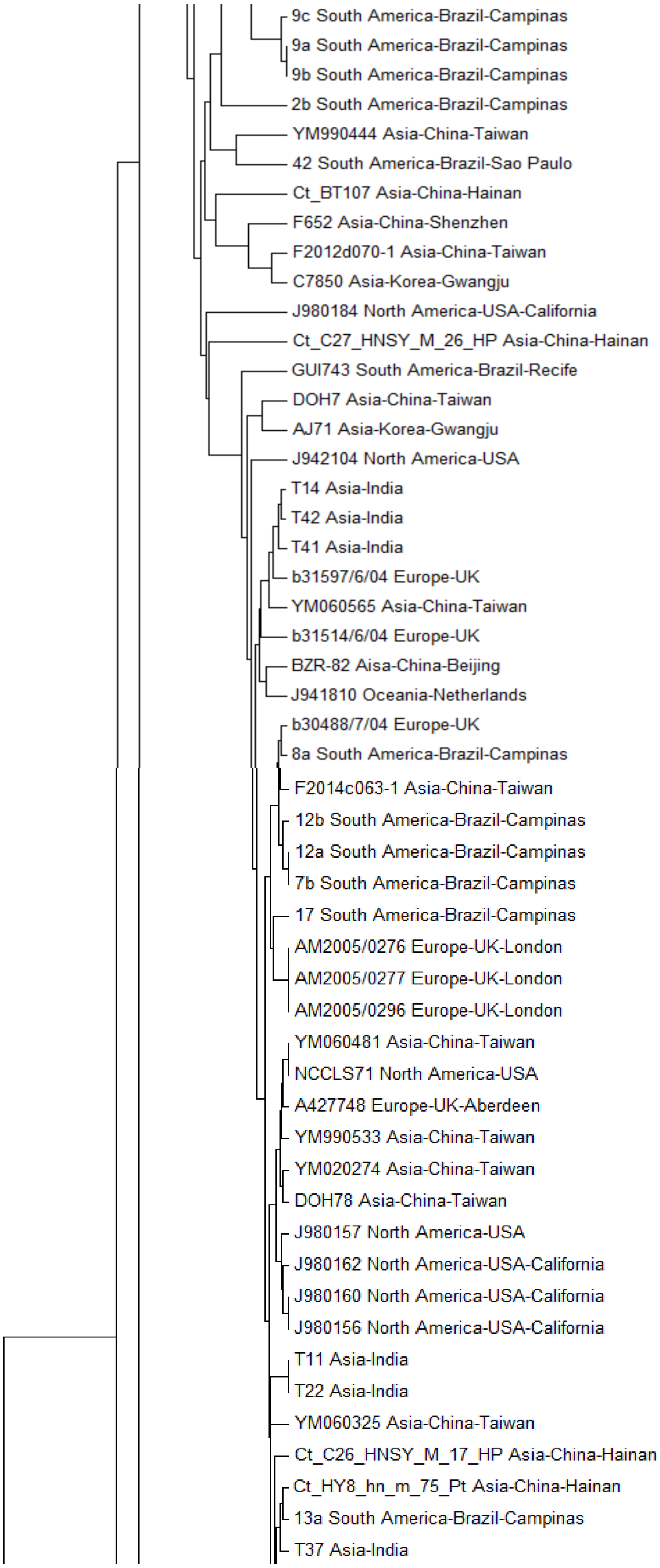
9c South America-Brazil-Campinas
9a South America-Brazil-Campinas
9b South America-Brazil-Campinas
2b South America-Brazil-Campinas
YM990444 Asia-China-Taiwan
42 South America-Brazil-Sao Paulo
Ct_BT107 Asia-China-Hainan
F652 Asia-China-Shenzhen
F2012d070-1 Asia-China-Taiwan
C7850 Asia-Korea-Gwangju
J980184 North America-USA-California
Ct_C27_HNSY_M_26_HP Asia-China-Hainan
GUI743 South America-Brazil-Recife
DOH7 Asia-China-Taiwan
AJ71 Asia-Korea-Gwangju
J942104 North America-USA
T14 Asia-India
T42 Asia-India
T41 Asia-India
b31597/6/04 Europe-UK
YM060565 Asia-China-Taiwan
b31514/6/04 Europe-UK
BZR-82 Aisa-China-Beijing
J941810 Oceania-Netherlands
b30488/7/04 Europe-UK
8a South America-Brazil-Campinas
F2014c063-1 Asia-China-Taiwan
12b South America-Brazil-Campinas
12a South America-Brazil-Campinas
7b South America-Brazil-Campinas
17 South America-Brazil-Campinas
AM2005/0276 Europe-UK-London
AM2005/0277 Europe-UK-London
AM2005/0296 Europe-UK-London
YM060481 Asia-China-Taiwan
NCCLS71 North America-USA
A427748 Europe-UK-Aberdeen
YM990533 Asia-China-Taiwan
YM020274 Asia-China-Taiwan
DOH78 Asia-China-Taiwan
J980157 North America-USA
J980162 North America-USA-California
J980160 North America-USA-California
J980156 North America-USA-California
T11 Asia-India
T22 Asia-India
YM060325 Asia-China-Taiwan
Ct_C26_HNSY_M_17_HP Asia-China-Hainan
Ct_HY8_hn_m_75_Pt Asia-China-Hainan
13a South America-Brazil-Campinas
T37 Asia-India

continued

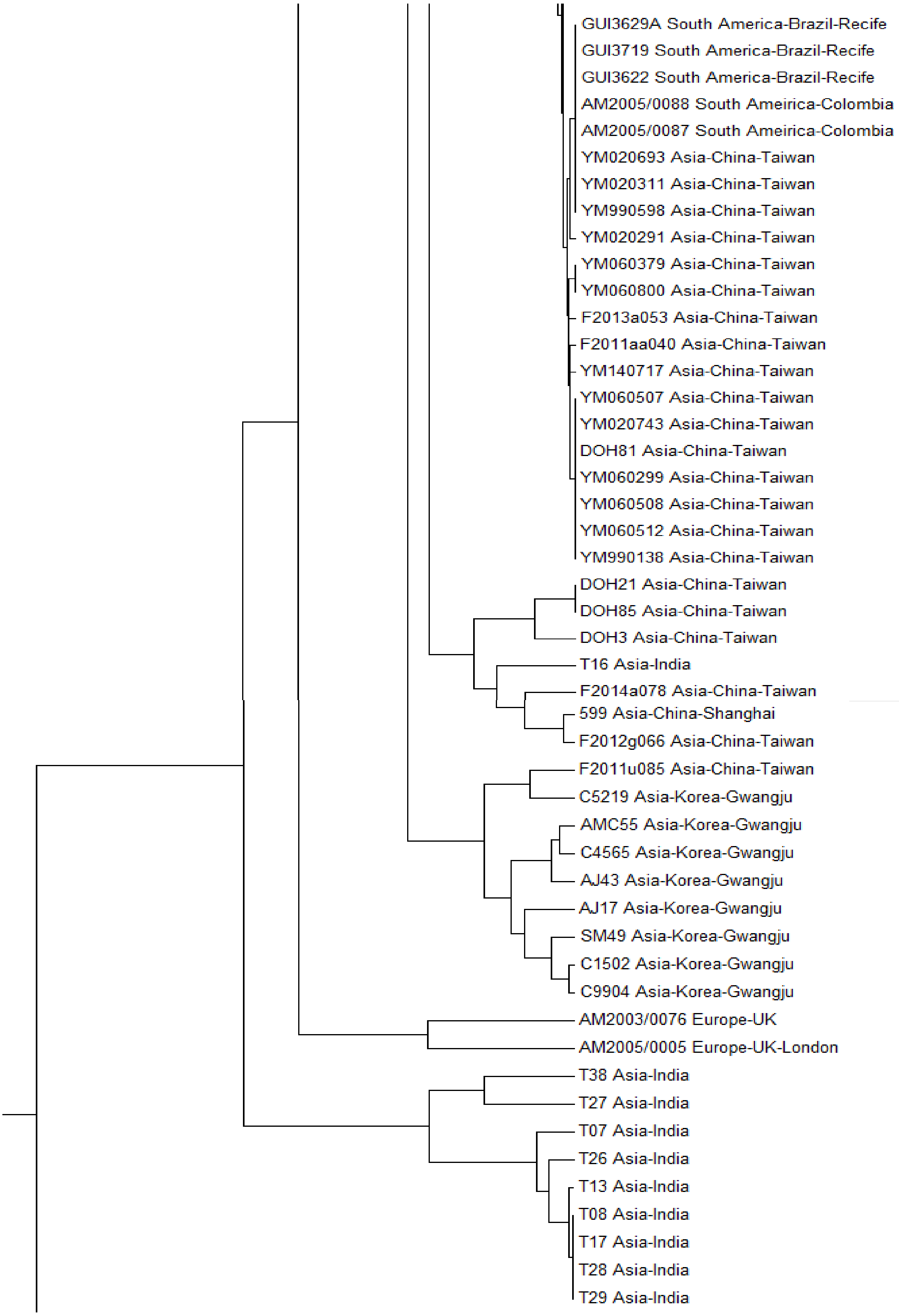
GUI3629A South America-Brazil-Recife
GUI3719 South America-Brazil-Recife
GUI3622 South America-Brazil-Recife
AM2005/0088 South Ameirica-Colombia
AM2005/0087 South Ameirica-Colombia
YM020693 Asia-China-Taiwan
YM020311 Asia-China-Taiwan
YM990598 Asia-China-Taiwan
YM020291 Asia-China-Taiwan
YM060379 Asia-China-Taiwan
YM060800 Asia-China-Taiwan
F2013a053 Asia-China-Taiwan
F2011aa040 Asia-China-Taiwan
YM140717 Asia-China-Taiwan
YM060507 Asia-China-Taiwan
YM020743 Asia-China-Taiwan
DOH81 Asia-China-Taiwan
YM060299 Asia-China-Taiwan
YM060508 Asia-China-Taiwan
YM060512 Asia-China-Taiwan
YM990138 Asia-China-Taiwan
DOH21 Asia-China-Taiwan
DOH85 Asia-China-Taiwan
DOH3 Asia-China-Taiwan
T16 Asia-India
F2014a078 Asia-China-Taiwan
599 Asia-China-Shanghai
F2012g066 Asia-China-Taiwan
F2011u085 Asia-China-Taiwan
C5219 Asia-Korea-Gwangju
AMC55 Asia-Korea-Gwangju
C4565 Asia-Korea-Gwangju
AJ43 Asia-Korea-Gwangju
AJ17 Asia-Korea-Gwangju
SM49 Asia-Korea-Gwangju
C1502 Asia-Korea-Gwangju
C9904 Asia-Korea-Gwangju
AM2003/0076 Europe-UK
AM2005/0005 Europe-UK-London
T38 Asia-India
T27 Asia-India
T07 Asia-India
T26 Asia-India
T13 Asia-India
T08 Asia-India
T17 Asia-India
T28 Asia-India
T29 Asia-India

continued

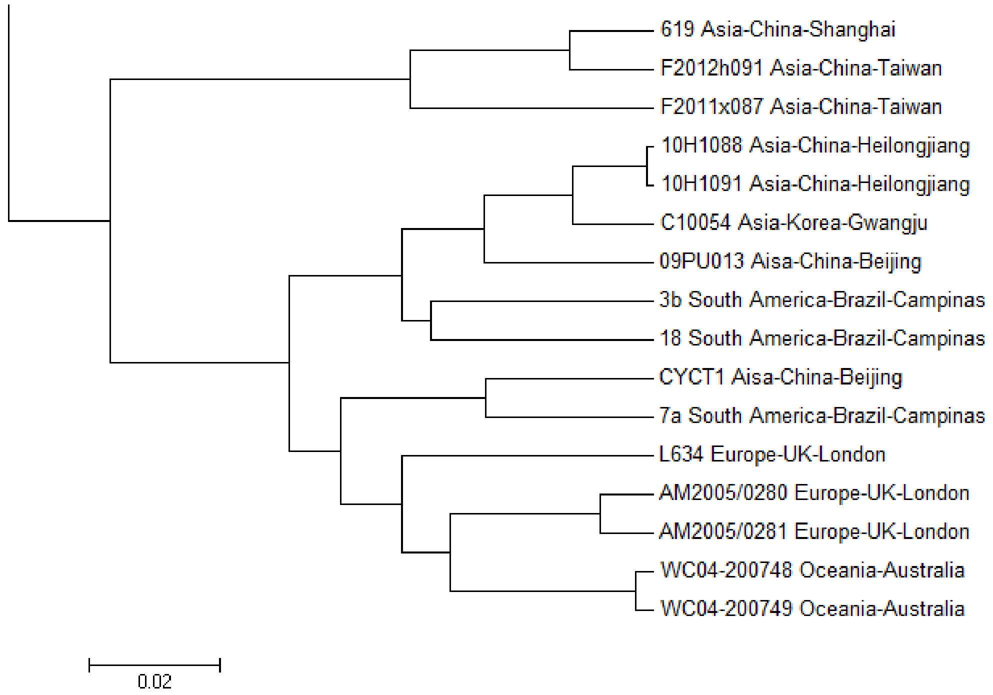

619 Asia-China-Shanghai
F2012h091 Asia-China-Taiwan
F2011x087 Asia-China-Taiwan
10H1088 Asia-China-Heilongjiang
10H1091 Asia-China-Heilongjiang
C10054 Asia-Korea-Gwangju
09PU013 Aisa-China-Beijing
3b South America-Brazil-Campinas
18 South America-Brazil-Campinas
CYCT1 Aisa-China-Beijing
7a South America-Brazil-Campinas
L634 Europe-UK-London
AM2005/0280 Europe-UK-London
AM2005/0281 Europe-UK-London
WC04-200748 Oceania-Australia
WC04-200749 Oceania-Australia
0.02
